# CryoEM-sampling of metastable conformations appearing in cofactor-ligand association and catalysis of glutamate dehydrogenase

**DOI:** 10.1038/s41598-024-61793-x

**Published:** 2024-05-15

**Authors:** Taiki Wakabayashi, Mao Oide, Masayoshi Nakasako

**Affiliations:** 1 Department of Physics, Faculty of Science and Technology, Keio University, 3-14-1 Hiyoshi, Kohoko-Ku, Yokohama, Kanagawa 223-8522 Japan; 2 RIKEN SPring-8 Center, 1-1-1 Kouto, Sayo-Cho, Sayo-Gun, Hyogo 679-5148 Japan; 3 PRESTO, Japan Science and Technology Agency, Chiyoda-Ku, Tokyo, 102-0076 Japan; 4 Protein Research Institute, Osaka University, Yamadaoka, Suita, Osaka 565-0871 Japan

**Keywords:** Biophysics, Structural biology

## Abstract

Kinetic aspects of enzymatic reactions are described by equations based on the Michaelis–Menten theory for the initial stage. However, the kinetic parameters provide little information on the atomic mechanism of the reaction. In this study, we analyzed structures of glutamate dehydrogenase in the initial and steady stages of the reaction using cryoEM at near-atomic resolution. In the initial stage, four metastable conformations displayed different domain motions and cofactor/ligand association modes. The most striking finding was that the enzyme-cofactor-substrate complex, treated as a single state in the enzyme kinetic theory, comprised at least three different metastable conformations. In the steady stage, seven conformations, including derivatives from the four conformations in the initial stage, made the reaction pathway complicated. Based on the visualized conformations, we discussed stage-dependent pathways to illustrate the dynamics of the enzyme in action.

## Introduction

Enzymes catalyze chemical reactions upon the formation of enzyme-ligand complexes. The kinetic behavior of enzymes in the initial stages of the reactions are described by a set of equations and several parameters representing the association/dissociation of ligands and catalysis^1^. Such kinetic theory assumes the enzyme–substrate complex as a single state. However, when spatiotemporal dynamics is present within the state, this assumption may be broken down, and it is difficult to deduce the dynamics only using the kinetic parameters. In fact, a series of infrared spectroscopic studies on lactate dehydrogenase demonstrated that the Michaelis complex was an ensemble of heterogeneous conformations in the probed local regions^2,3^. Further investigation into the whole conformational dynamics of enzymes in action are still under progress. Therefore, we are curious on the conformational changes over the enzymes during the association with the ligand molecules, the catalytic reaction and the dissociation of product molecules.

Crystal structures of enzyme-ligand analogue complexes provide the atomic details of the molecular interactions necessary for the enzymatic reactions^4^ and are helpful for discussing the mechanisms underlying the conformational changes of enzymes, such as lock-and-key, induced fit, conformational sampling, and linear response^5–8^. Although the penetration pathways of small molecules into the reaction centers of enzymes were studied for proteins in crystalline state^9–11^, the molecular contacts necessary to maintain the crystalline arrangements may suppress large conformational changes of enzymes that naturally occur in solution during the association with cofactor/ligand molecules and catalysis.

In contrast, cryoEM, that analyzes the structures of frozen-hydrated enzymes, can visualize the conformational variations of enzymes in action^12^. In particular, the focused classification^13^ is a powerful tool for extracting metastable conformations at a near-atomic resolution, as demonstrated in studies on the pathways and free-energy landscapes of conformational changes in supramolecular complexes^14–16^. When studying the enzyme-ligand association process using cryoEM, good targets are multi-domain enzymes that frequently exhibit large-scale motions of domains between the unliganded and liganded states and spontaneously even in the unliganded state^17^.

**Figure 1:**
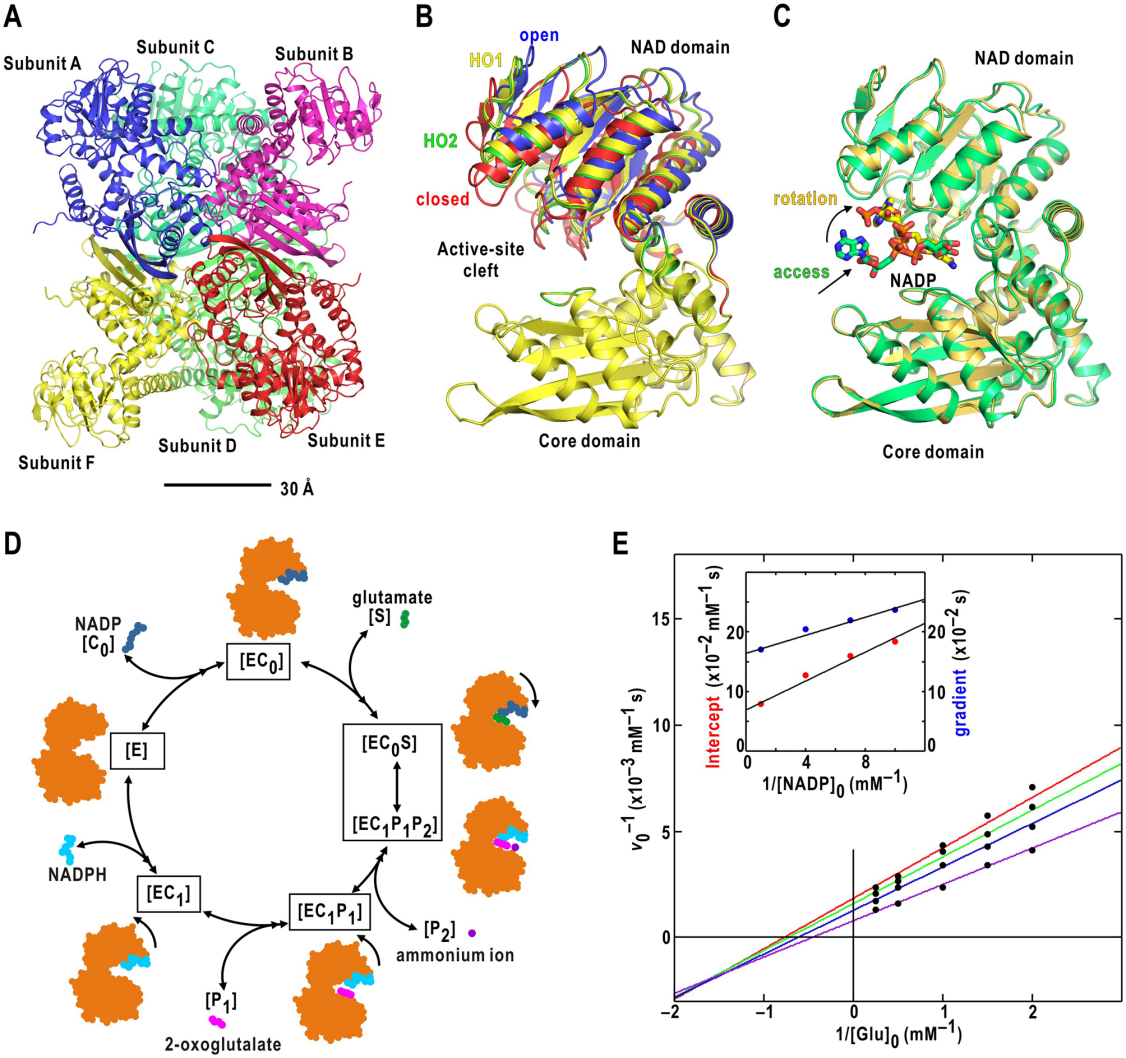
Structures and enzymatic reaction of glutamate dehydrogenase. (**A**) Crystal structure of unliganded glutamate dehydrogenase (GDH) hexamer from *Thermococcus profundus* (the PDB accession code: 1EUZ)^21^. (**B**) Four metastable NAD-domain motions in unliganded state as revealed by cryoEM^22^. The PDB accession codes for the open (blue-colored model), half-open 1(HO1) (yellow), half-open 2 (HO2) (green) and closed (red) conformations are 6JNA, 6JNC, 6JND and 6JN9, respectively. (**C**) Two representative cryoEM structures of the nonproductive GDH-NADP complex^26^. The PDB accession codes are 8HIQ (yellow-colored model) and 8HJQ (green), respectively. The panels (**A**) and (**B**) were drawn using *PyMOL*^65^. (**D**) Schematic diagram of the ordered bi reaction of GDH. *E*, *C*_0_, *S*_,_ *C*_1_, *P*_1_, and *P*_2_ represent GDH, NADP, glutamate, NADPH, 2-oxoglutarate, and ammonium ion, respectively. (**E**) Lineweaver–Burk (L–B) plot for the ordered bi reaction of GDH at five glutamate (0.50, 0.67, 1.00, 2.00 and 4.00 mM) and four NADP concentrations (0.10 [red regression line], 0.14 [green], 0.25 [blue], and 1.00 mM [purple]). The inset panel shows the linear dependences of the intercept and gradient values in the L-B plot on the reciprocal of NADP concentration. The regression lines were calculated using least-square fitting to the data points.

We have been studying the structure and dynamics of a homo-hexameric enzyme, glutamate dehydrogenase (GDH)^18^ (Fig. 1A). Each subunit with molecular weight of 46–50 k folds into the core domain for the hexamer formation and the NAD domain to bind a cofactor molecule. A large active-site cleft is situated between the domains^18^. GDH existing in both prokaryotes and eukaryotic cells catalyzes the oxidative deamination reaction to convert glutamate to 2-oxoglutarate and ammonia in the presence of the cofactor, such as, nicotinamide adenine dinucleotide phosphate in the oxidized form (NADP). Then, GDH acts as, for instance, a recycler of the neurotransmitter, glutamate^19^ and as a coordinator in cell division^20^ by reversibly.

Our previous structural studies on unliganded GDH from *Thermococcus profundus* revealed four metastable conformations^21,22^, designated as open, half-open1 (HO1), half-open2 (HO2), and closed in the NAD-domain motion to spontaneously and stochastically open/close the cleft with the free energy differences of 0.07–0.12 kcal/mol at 300 K^22^ (Fig. 1B). In this regard, the crystal structure and molecular dynamics (MD) simulation suggested that the dissociation and association of a few hydration water molecules at the depth of the cleft are likely important to regulate the NAD-domain motion^21,23–25^. In addition, the structures of nonproductive GDH-NADP complex (Fig. 1C) suggested a NAD-domain conformation suitable for NADP-binding and approaching pathways of NADP to the final-binding site^26^.

The enzymatic reaction of *T. profundus* GDH is described by the ordered bi ter scheme^27^ under the assumption that the GDH-cofactor complex is formed before the substrate-binding (Fig. 1D). The Lineweaver–Burk (L-B) plot for the reaction kinetics in the initial stage experimentally confirmed the scheme (Fig. 1E and Supplementary Table S1). Our previous studies revealed that both the ligand-free ([*E*]) and GDH-NADP complex ([*EC*_0_]) states displayed multiple NAD-domain conformations (Fig. 1B,C)^21,22,26^. In this study, we extended the previous studies over the enzymatic reaction, and visualized NAD-domain conformations appearing in the initial and steady stages using cryoEM, and examined whether each state in the reaction scheme was a mixture of heterogeneous and metastable conformations. Based on the observed conformations, we discuss stage-dependent reaction pathways.

## Results

### Overall structures

**Figure 2:**
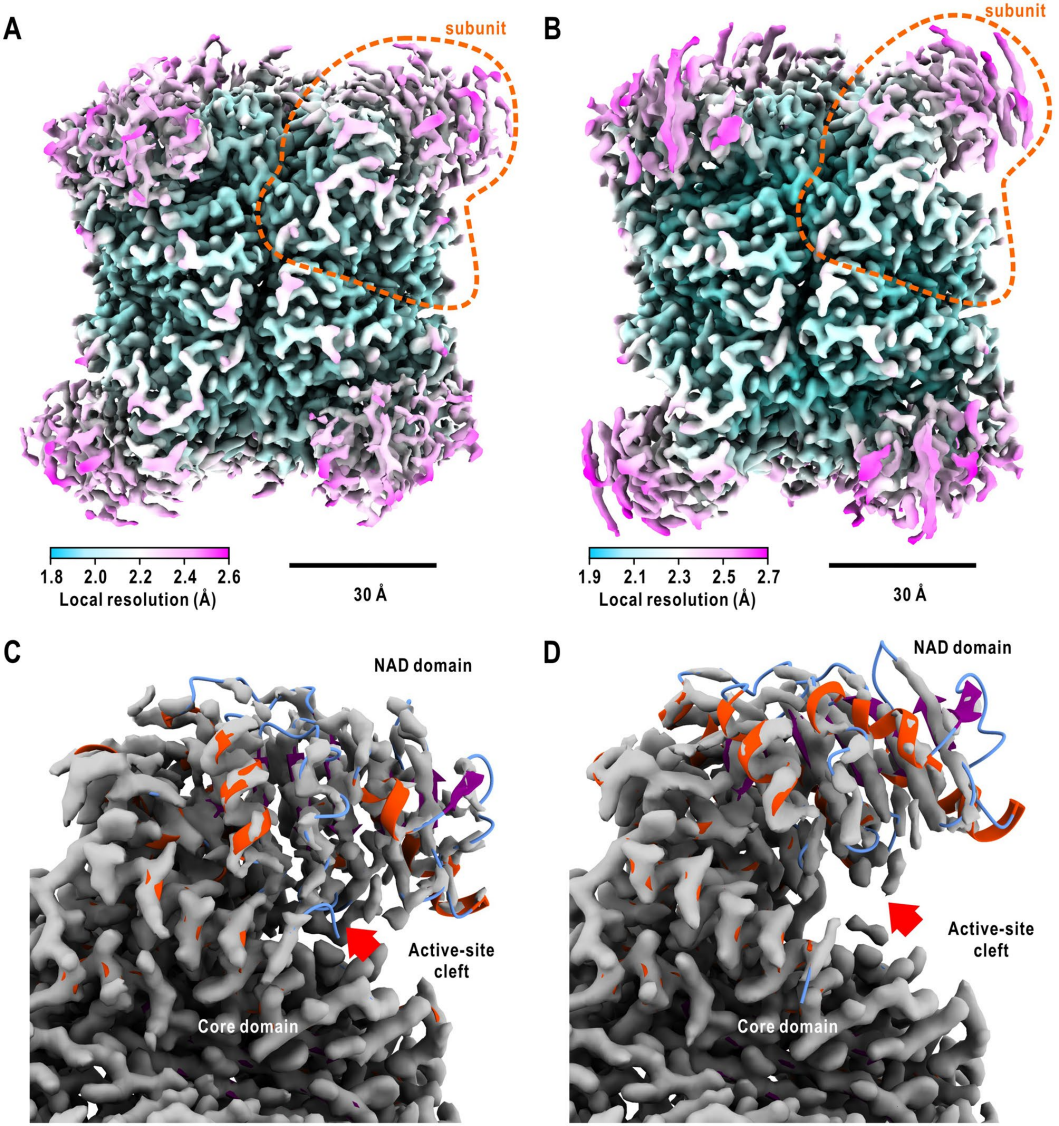
Structures of GDH hexamers in the initial and steady stages. Three-dimensional potential maps of GDH hexamers assumed the D3 symmetry in the initial (**A**) and steady (**B**) stages, respectively. Each map was contoured at 2 standard deviation level from the average and colored according to the local resolution as indicated at the bottom left of each panel. Superimposition of a subunit in the crystal structure onto a subunit of the D3 maps in the initial (**C**) and steady (**D**) stages after optimal overlap of the core-domain model with the map. In the ribbon model of the crystal structure, α-helices, β-strands and loops are colored red, purple and cyan, respectively. The red arrows indicate maps assignable to cofactor molecules. All panels were prepared using *UCSF ChimeraX*^66^.

GDH images in the initial stage were collected from a GDH-NADP-glutamate solution flash-cooled 15-s after the mixing at 277 K. Under assuming the D3-symmetry in the hexamer, the three-dimensional potential map (so-called consensus map) of GDH hexamer was reconstructed at a resolution of 2.2 Å (Fig. 2A and Supplementary Fig. S1). The dimensions of the map were more compact than those of the unliganded state^21,22^, suggesting that subunits with closed active-site clefts were predominant.

For the analysis of the steady stage, we flash-cooled a GDH-NADP-glutamate solution 3600-s after the mixing at 293 K. The consensus map reconstructed at a resolution of 2.3 Å had dimensions larger than those of the initial stage but slightly smaller than those of the unliganded state (Fig. 2B and Supplementary Fig. S2). This result was consistent with the radius of gyration value in the steady stage (42.2 ± 0.1 Å), which was slightly smaller than that of the unliganded state (43.2 ± 0.1 Å) (Supplementary Note 1 and Fig. S3).

In the consensus map of each stage (Fig. 2C,D), the core-domain map displayed a local resolution higher than 2.0 Å and was consistent with the crystal structure model. In contrast, model building for cofactor/ligand molecules in the active-site cleft was difficult due to the blurred NAD-domain map. Therefore, the blurred region was interpreted as a mixture of heterogeneous NAD-domain conformations and cofactor/ligand-binding modes.

### NAD-domain conformations

Two-step focused classification was carried out for the volume, which included the NAD domain, a part of the core domain, and the active-site cleft (Supplementary Note 2 and Fig. S4). In the first step, the D3 consensus-map was separated into several classes with respect to the NAD-domain conformations. The second classification step divided each class into subclasses, predominantly with respect to the maps of the active-site cleft and was helpful to exclude inappropriate images. As classification for noisy and low contrast images is better to be performed multiple times^28^, we compared maps from independently performed classification trials for each stage. Then, after evaluating the quality and reproducibility of maps (Supplementary Note 2 and Figs. S5‒S10), we selected the best trial providing maps most suitable to build structural models and cofactor/ligand molecules (Supplementary Figs. S11‒S12 and Table S2).

**Figure 3:**
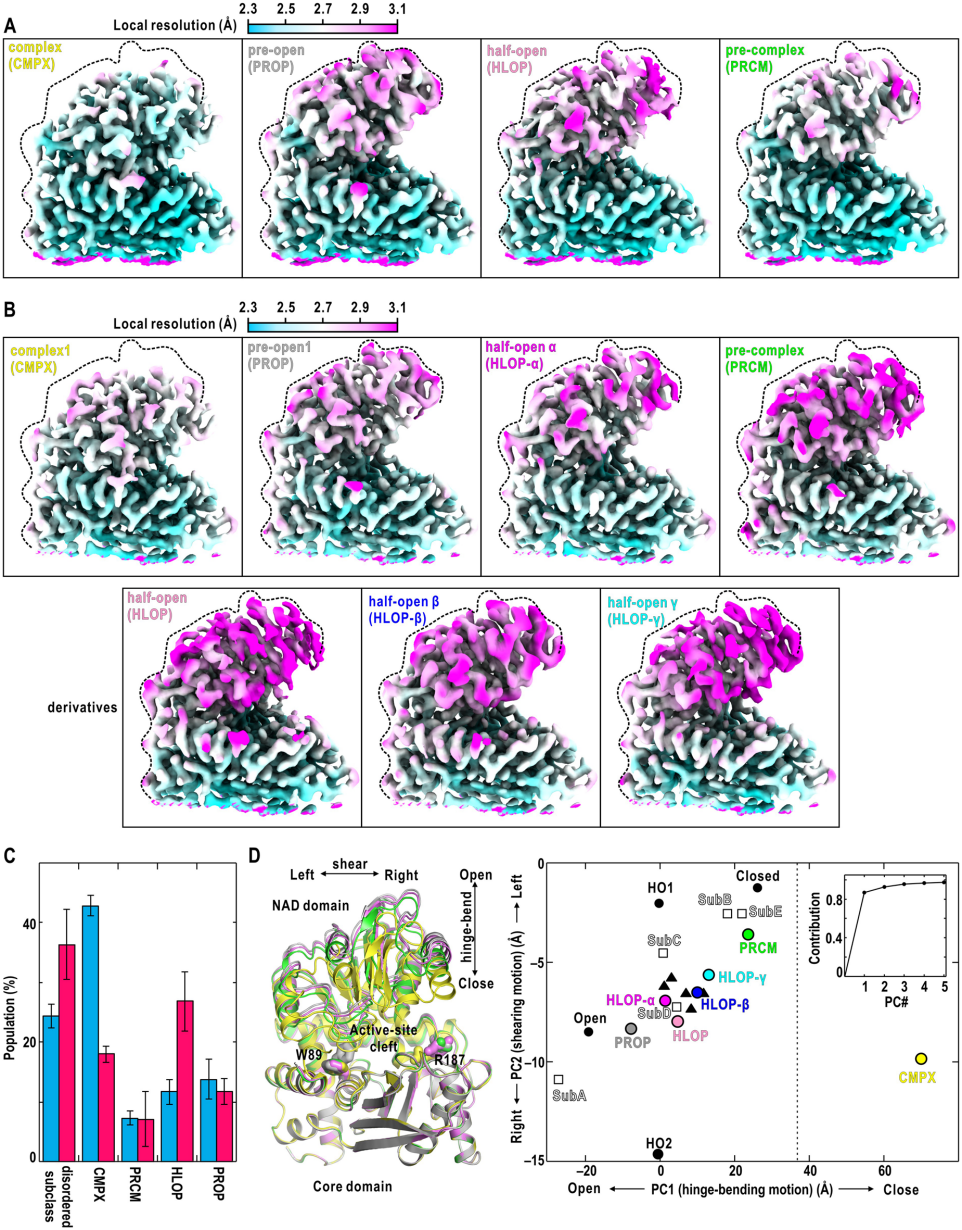
NAD-domain conformations in the initial and steady stages. Refined potential maps provided by the selected classification trials. Depicted are four conformations in the initial stage (**A**) and seven in the steady stage (**B**). In panel (**B**), three conformations, which are derivatives of HLOP in the initial stage, are also shown in the second row. The maps are contoured at 2 standard deviation level from the average and colored according to the local resolution shown at the top of the panel. The dashed line in each panel indicates the rough edge of the NAD domain in PROP. The label at the top of each panel indicates the name of the conformation and its abbreviation in parentheses. (**C**) Averaged populations of images in the “disordered map”, CMPX, PRCM, HLOP and PROP in five classification trials for the initial stage (blue bars) and those for the steady stage (magenta bars). Percentages were calculated as the ratio of each category for the total number of images used in the classification. Bars are the standard deviations. The detailed results for trials are shown in Supplementary Note 2 and Fig. S8. (**D**) The left panel depicts the variation in NAD-domain motions among PROP (gray), HLOP (pink), PRCM (green), and CMPX (yellow) in the initial stage. The arrows indicate the approximate directions of the hinge-bending and shearing motions. The right panel presents the distribution of the representative NAD-domain conformations observed in the initial and steady stages (colored circles) on the plane spanned by two vectors. The two vectors represent the hinge-bending and shearing motions, respectively, and contribute to more than 93% of the NAD-domain motion (inset panel). The dashed line indicates the approximate limit of hinge-bending motion sampled in the previous MD simulation for unliganded GDH^31^. The reference structures are four NAD conformations from cryoEM in Fig. 1B (black circles)^22^ and five subunits of the crystal structure in Fig. 1A (open squares)^21^ both in the unliganded state. The black triangles indicate the five nonproductive GDH-NADP complexes in Fig. 1B ^26^. Panels (**A**) and (**B**) were prepared using *UCSF Chimera X*^66^, and *PyMOL*^65^ was used for the left panel of (**D**).

Regarding the initial stage, the D3 map was a mixture of four NAD-domain conformations (Fig. 3A). The most popular maps displayed NAD-domain conformation with the active-site cleft closest among the four. The structural models refined for the maps were almost the same as indicated by root-mean-square differences (r.m.s.d.) smaller than 0.15 Å for all mainchain atoms. In the most closed NAD conformation, the jaw of the NAD domain contacted with that of the core domain, and was superimposable onto the crystal structure of the nonproductive complex of *Corynebacterium glutamicum* GDH, NADP, and 2-oxoglutarate^29^ (Supplementary Fig. S13). Therefore, the most closed NAD-conformation was designated as “complex” (abbreviated as CMPX).

The NAD-domain conformation with the most open cleft among the four was designated “pre-open” (PROP), because the active-site cleft was narrower than those of the open conformation in the unliganded states^21,22^. From PROP to CMPX, the tip of the NAD domain travels by 12 Å, and then the volume of the active-site cleft calculated using *MoloVol*^30^ will be reduced by approximately 1900 Å^3^. This volume change corresponds to the exclusion of more than 60 water molecules from the active-site cleft. The other two NAD-domain conformations were in between CMPX and PROP. The NAD-domain conformation similar to the GDH-NADP complex was designated “half-open” (HLOP). Another was designated “pre-complex” (PRCM), because the active-site cleft was still wider than CMPX. From PRCM to CMPX, the tip of the NAD domain travels by 8 Å.

The D3-map of the steady stage was a mixture of seven NAD-domain conformations (Fig. 3B). The NAD-domain conformations with the most closed active-site cleft were superimposable onto those of CMPX in the initial stage with the r.m.s.d. values smaller than 0.15 Å for all mainchain atoms. Three NAD conformations were superimposable onto PROP, HLOP, and PRCM in the initial stage, respectively, with the r.m.s.d. values smaller than 0.23 Å for all mainchain atoms. Therefore, the CMPX, PRCM, HLOP, and PROP conformations commonly existed in both stages. The other three NAD-domain conformations observed in the steady stage only were slightly different from that of HLOP. Then, the three conformations were assumed as derivatives from HLOP and designated half-open α (HLOP-α), HLOP-β, and HLOP-γ, respectively, in the order of the wide to narrow active-site cleft.

The populations of images in the PROP, HLOP, PRCM and CMPX conformations were different between the initial and steady stage (Fig. 3C and Supplementary Fig. S8). The population of CMPX in the initial stage (42.8% of used images) was 2.4-fold of that in the steady stage (18.0%), and contributed to make the D3-map of the hexamer in the initial stage more compact than that in the steady (Fig. 2A). In contrast, the population of HLOP including the derivatives in the steady stage (26.8%) was approximately 2.3-fold of that in the initial (11.7%). Those of PROP (13.1% in the initial and 11.7% in the steady) and PRCM (7.3% in the initial and 7.1% in the steady) were comparable between the two stages. These findings indicated that the populations of the CMPX and HLOP conformations were stage-dependent.

### Characterization of NAD-conformations

NAD-domain conformations are predominantly characterized by hinge-bending and shearing motions relative to the core domain as reported in our MD simulation and cryoEM studies on the unliganded state^22,24^. In Fig. 3D, the observed NAD-domain conformations were projected onto the plane spanned by two vectors, which were obtained by principal component analysis for the previous MD trajectory^31^ and represented the hinge-bending and the shearing motions. CMPX displayed a magnitude of the hinge-bending motion twice that of the closed conformation in the unliganded state and was located out of the area sampled by the MD simulation for the unliganded state, suggesting that the CMPX conformation was probably induced by the association of the cofactor and ligand molecules. In contrast, the magnitudes of the hinge-bending and shearing motions of PROP, HLOP, and PRCM were within the sampled area (Fig. 3D).

PROP was located between the open conformations in the unliganded state^21^ and the nonproductive GDH-NADP complex^26^. The magnitudes of the hinge-bending motions in HLOP, HLOP-α, HLOP-β, and HLOP-γ conformations were similar to those of the HO-1 and HO-2 conformations in the unliganded state^22^, but those of the shearing motions were in between HO-1 and HO-2. Regarding the PRCM conformation, the magnitude of hinge-bending motion was comparable to that of the closed conformation in the unliganded state. From PRCM to CMPX, the shearing motion occurred in the opposite direction to that from PROP to PRCM.

It should be noted that Trp89 and Arg187 sidechains, which were located at the edges of the active-site cleft, displayed alternative conformations in concert with the NAD-domain motion^21,22^ (Supplementary Figs. S14‒S15). The Trp89 sidechain took two conformers in subclasses of PROP, HLOP, and HLOP-α, and the occupancies of the two conformers depended on the width of the cleft. The Arg187 sidechain took two conformers in HLOP and PRCM, and the tip of one conformer pointed toward the center of the cleft.

### Structure of GDH-cofactor-ligand complex

**Figure 4:**
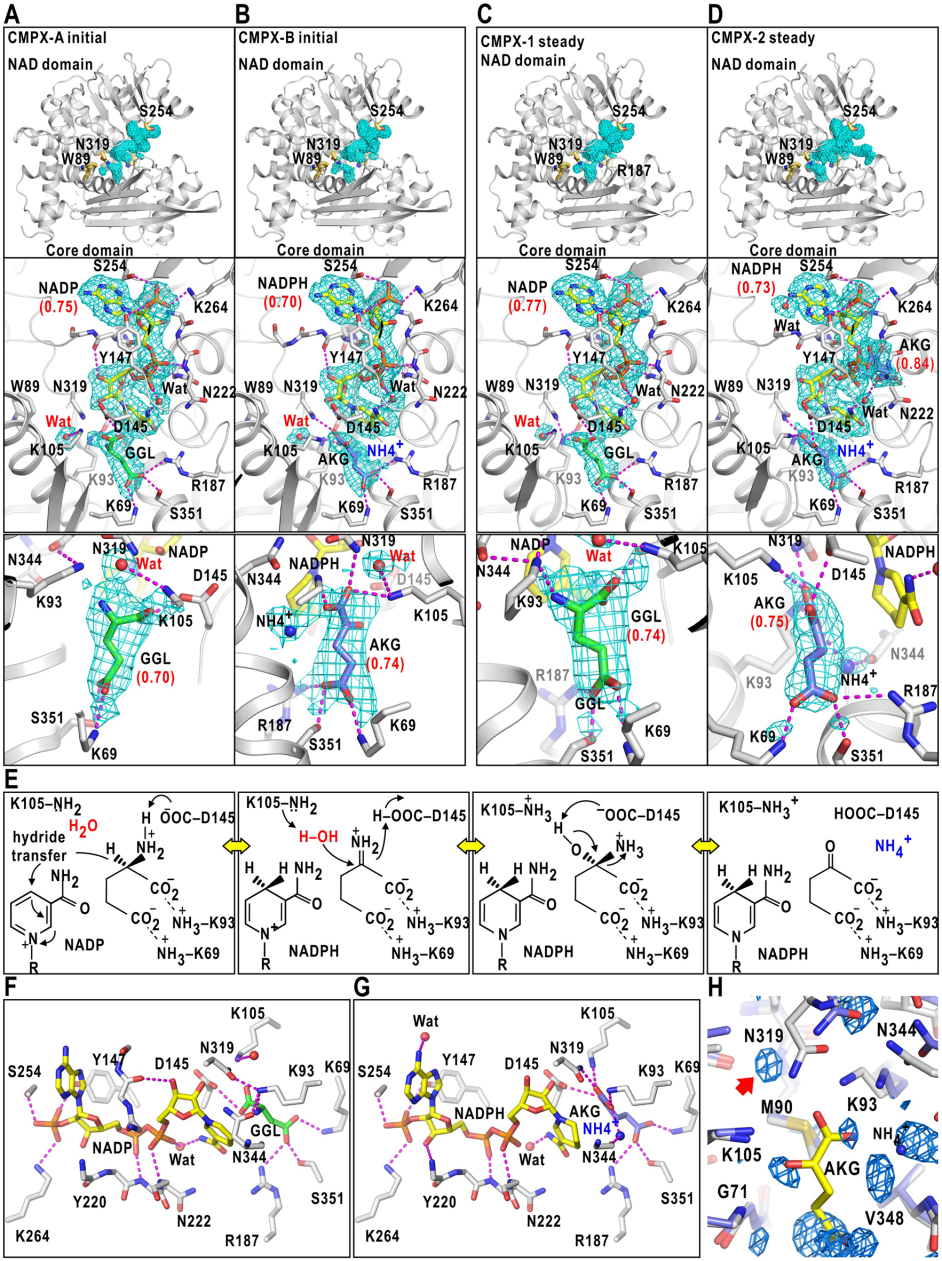
Structures of GDH-cofactor-substrate/product complex. Potential maps of the cofactor and substrate molecules of CMPX-A (**A**) and CMPX-B (**B**) in the initial stage, and those of CMPX-1 (**C**) and CMPX-2 (**D**) in the steady stage. In each, the upper panel displays the locations of the cofactor/ligand maps in the active-site cleft of a subunit with the sidechains of landmark residues. The middle panel is the magnified view of the upper panel with sidechains engaged in the binding (stick models) and possible hydrogen bonds with a donor–acceptor distance shorter than 3.4 Å (magenta dashed lines). The bottom panel presents a magnified view of the maps in the ligand-binding pocket with a ligand model fitted to the map. Glutamate and 2-oxoglutarate molecules are labeled as GGL and AKG, respectively. The maps were contoured at 2–3 standard deviation levels from the average. The *Q*-scores^33^ of cofactor, ligand and solvent molecules are shown in the parentheses below the abbreviated molecular names (Supplementary Note 3 and Tables S3‒S4). (**E**) Schematic of the probable catalytic reaction expected in the ligand-binding pocket of CMPX. Panels (**F**) and (**G**) illustrate hydrogen bonds with a donor–acceptor distance shorter than 3.4 Å in the GDH-NADP-glutamate complex and in the GDH-NADPH-2-oxoglutalate complex, respectively. (**H**) Distribution of hydration water molecules (maps in blue mesh) in the ligand-binding pocket of unliganded GDH^19,29^. Most of the hydration water molecules were excluded by the ligand molecule. The stick models are CMPX-2 (white-colored carbon atoms) optimally superimposed onto subunit E (blue) of the crystal structure (the PDB accession code: 1EUZ)^21^. The red arrow indicates the map of a water molecule between Lys105 and Asn319. The mesh is the omit-difference Fourier map of hydration water molecules calculated at a resolution of 1.8 Å and contoured at 3.5 standard deviation level from the average. Panels (**A**)–(**D**) and (**F**)–(**H**) were drawn using *PyMOL*^65^.

In each CMPX of both stages, a Y-shaped map attached to the NAD domain was modeled as either of NADP or NADPH molecule (Fig. 4A‒D, Supplementary Note 3, Table S3). In addition, a map assignable to substrate (glutamate) or product (2-oxoglutarate) molecule occupied the ligand-binding pocket, that was formed by the sidechains of Lys69, Lys93, Lys105, Arg187, and Ser351 of the active-site cleft. The tip of the Arg187 sidechain was moved by 7 Å from the position in the unliganded state (Supplementary Fig. S13C). As the active-site cleft in association with the cofactor and ligand molecules was closed and suitable for the catalytic reaction, each of the CMPX maps was assigned to either of [*EC*_0_*S*], [*EC*_1_*P*_1_*P*_2_] (Fig. 1D), or their mixture.

Assuming a probable scheme regarding the reversible conversion of glutamate to 2-oxoglutarate (Fig. 4E), one hydration water molecule near the Lys105 sidechain is necessary to proceed with the first and second steps of [*EC*_0_*S*], and one ammonium ion is produced near 2-oxoglutarate at [*EC*_1_*P*_1_*P*_2_]. In addition, 2-oxoglutarate molecule has a planar *sp*^2^-hybridization geometry of the carboxylate group, while glutamate molecule contains the Cα atom with the tetrahedral arms in the *sp*^3^-hybridization geometry. As the local resolution of the ligand-binding pocket was higher than 2.3 Å in some CMPX subclasses (Fig. 3A,B), we attempted to assign each CMPX map to [*EC*_0_*S*], [*EC*_1_*P*_1_*P*_2_], or their mixture by inspecting the fitness of the structural model of substrate/product molecule to the map (Fig. 4A‒D) and quantitatively evaluating the correlation scores^32,33^ between the maps and models (Supplementary Note 3, Table S4 and Fig. S17).

In a representative CMPX conformation of the initial stage (designated CMPX-A), the ligand map with a bulge toward Asn344 was better fitted by a glutamate molecule than by a 2-oxoglutarate (Fig. 4A). In addition, any map assignable to a solvent molecule was absent from a room between the ligand map and Asn344, while a map assignable to a hydration water molecule appeared between the sidechains of Lys105 and Asn319. Thus, the cofactor was modeled as NADP, and CMPX-A was assigned to [*EC*_0_*S*]. In another CMPX (CMPX-B), the ligand map displayed a planar shape of 2-oxoglutarate and a solvent map assignable to an ammonium ion in front of the carbonyl groups of the Asn344 mainchain (Fig. 4B). Therefore, this map corresponded to [*EC*_1_*P*_1_*P*_2_].

In the steady stage, we also assigned two CMPX (CMPX-1 in Fig. 4C and CMPX-2 in Fig. 4D) as [*EC*_0_*S*] and [*EC*_1_*P*_1_*P*_2_], respectively. In CMPX-2, another 2-oxoglutarate molecule attached to the flank of the pyrophosphate group of NADPH (middle panel of Fig. 4D).

The association modes of the cofactor molecules and the positions and orientations of the ligand molecules were almost the same in both stages. In both [*EC*_0_*S*] and [*EC*_1_*P*_1_*P*_2_], the cofactor molecule was fixed by nine hydrogen bonds with residues of the NAD domain (Fig. 4F,G). The glutamate molecule in [*EC*_0_*S*] was fixed by seven hydrogen bonds with the sidechains of the residues forming the ligand-binding pocket as well as the 2-oxoglutarate molecule in [*EC*_1_*P*_1_*P*_2_]. Hydrogen bonds between Asn319 of the NAD domain and Asp145/Lys93 of the core domain contributed to keep the closed conformation.

The cofactor binding induced small positional shifts from the unliganded state^21^ at the Gly221-Asn222 mainchain of the NAD domain by 0.4–0.7 Å and at the Lys93 sidechain in the ligand-binding pocket (Fig. 4H). In addition, a comparison of the ligand-binding pocket between CMPX and the unliganded crystal structure revealed that some hydration sites in the unliganded state were occupied by the cofactor and ligand molecules (Fig. 4H and Supplementary Fig. S18). One hydration water molecule necessary to proceed the first and second steps of the reaction was already present between Lys105 and Asn319 in the unliganded state.

### Variety in cofactor maps

**Figure 5:**
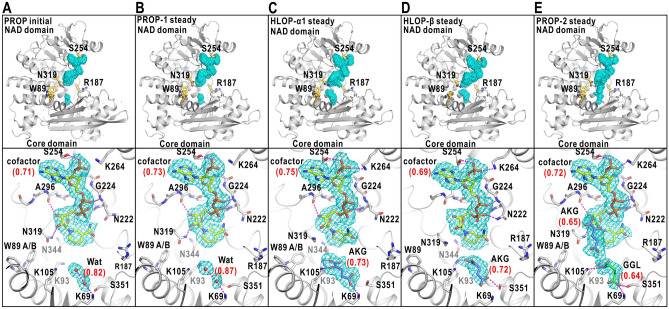
Structures of GDH-NADPH complex. Potential maps and structure models are presented for PROP in the initial stage (**A**), and PROP-1 (**B**), HLOP-α1 (**C**), PROP-γ (**D**), PROP-2 (**E**) in the steady stage. In all panels, the cofactor molecules are putatively modeled as NADPH and the *Q*-scores^33^ in parentheses are labeled to the cofactor, ligand and solvent molecules (Supplementary Note 3 and Tables S3‒S4). Each panel is illustrated in the manner of Fig. 4A–D. Magnified views of the maps of each ligand-binding pocket are presented in Supplementary Figs. S16‒S17. Panels were drawn using *PyMOL*^65^.

**Figure 6:**
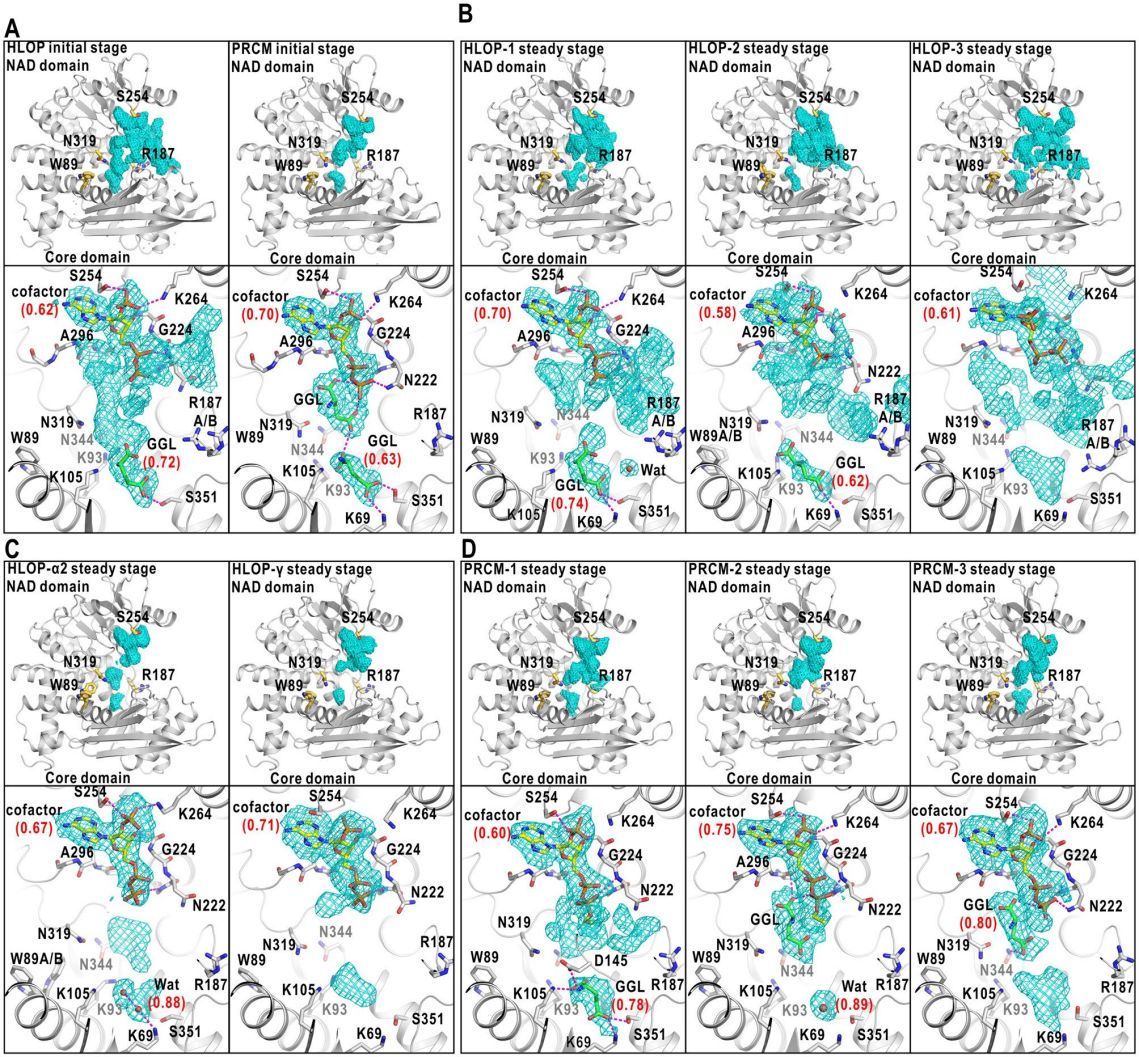
Structures of GDH-NADP complex. Potential maps and structural models in the ligand-binding pockets of HLOP and PRCM in the initial stage (**A**), HLOP-1, HLOP-2, HLOP-3 in the steady stage (**B**), HLOP-α2, HLOP-γ in the steady stage (**C**), and PRCM-1, PRCM-2, PRCM-3 in the steady stage (**D**). For each cofactor map, the adenosine-pyrophosphate group was modeled and the *Q*-scores^33^ in parentheses are labeled to the cofactor, ligand and solvent molecules (Supplementary Note 3 and Tables S3‒S4). Each panel is drawn in the manner of Fig. 4A–D. Magnified views of the maps and the fitted models in and around each ligand-binding pocket are presented in Supplementary Fig. S17B–E. Panels were drawn using *PyMOL*^65^.

In both stages, the adenosine-pyrophosphate groups of the cofactor molecules were visible in all maps and contacted with the NAD domains (Figs. 5 and 6, Supplementary Note 3, Tables S3‒S4 and Fig. S16). Regarding the initial stage, these results were consistent with the estimation from the dissociation constant of NADP (Supplementary Table S1) that more than 87% of GDH molecules was in association with cofactor molecules.

The maps of cofactor molecules were divided into two types. Maps of almost entire structures of the cofactor molecules were visible in PROP of both stages, and in one of HLOP-α (designated HLOP-α1), and HLOP-β of the steady stage (Fig. 5, Supplementary Note 3 and Table S3). As the ribose-nicotinamide group had the smaller number of interactions with the NAD domain than that of the adenosine-pyrophosphate group, the appearance of the map corresponding to the ribose-nicotinamide group implied its conformational rigidity in the bound cofactor molecule. In contrast, the ribose-nicotinamide groups were unclear or invisible in the maps of HLOP and PRCM of both stages and in one of HLOP-α (designated HLOP-α2) and HLOP-γ in the steady stage, probably due to the conformational disorder (Fig. 6, Supplementary Note 3, Table S3 and Figs. S16‒S17).

NADP has the C4N atom of the nicotinamide group in the *sp*^2^-hybridization, while the C4N atom of NADPH is in the *sp*^3^-hybridization. However, the local resolution of each map was insufficient to distinguish the structural difference between NADP and NADPH. The assignment of NADP or NADPH molecule to the map of each NAD-domain conformation will be described in the Discussion section.

### Molecules occupying the ligand-binding pocket

Next, we modeled ligand and/or solvent molecules to the maps in the ligand-binding pocket by inspecting the size and shape along with the correlation scores between the maps and models^32,33^. The details of the modeling are described in Supplementary Note 3, Table S4 and Fig. S17. In this analysis, we missed GDH-glutamate complex such as observed in *Clostridium symbiosum* GDH^34^, despite the stability for a long period of time to be crystallized.

In the initial stage, the map in PROP was modeled as two hydration water molecules (Fig. 5A). In HLOP, the map was modeled as a glutamate molecule (Fig. 6A). The disordered maps appearing at the mouth region of the active-site cleft could be interpreted as a cofactor and/or substrate molecules in various poses rushing into the cleft. In PRCM, one glutamate molecule occupied the ligand-binding pocket (Fig. 6A). At the flank of the pyrophosphate group of NADP, a map was modeled as another glutamate molecule (Fig. 6A, Supplementary Note 3, Tables S3‒S4 and Figs. S16‒S17). Disordered maps were absent from the active-site cleft of PRCM narrower than that of HLOP.

In the steady stage, the maps occupying the ligand-binding pockets were in variety even in the same NAD-domain conformations (Figs. 5, 6). In one of PROP designated PROP-1, the map in the pocket was modeled as two hydration water molecules (Fig. 5B). In another PROP (PROP-2) (Fig. 5E), one glutamate molecule occupied the binding pocket, and one 2-oxoglutarate molecule was associated with the flank of the pyrophosphate group of the cofactor molecule. The maps in one of HLOP-α (HLOP-α1) and HLOP-β were modeled as a 2-oxoglutarate molecule (Fig. 5C,D).

In two of HLOP (HLOP-1 and HLOP-2) and one of PRCM (PRCM-1), one glutamate molecule was modeled for the map in the pockets (Fig. 6B,D). One of HLOP-α (HLOP-α2) and one of PRCM (PRCM-2) had maps interpretable as two and one hydration water molecules, respectively (Fig. 6C,D). In contrast, in the third HLOP (HLOP-3), HLOP-γ, and one of PRCM (PRCM-3), the maps in and around the ligand-binding pocket were difficult to be modeled as a single ligand or water molecule (Fig. 6B,C), probably due to the positional disorder of molecules and/or in mixture even after the second classification.

In addition to the ligand-binding pocket, maps at the flank of the pyrophosphate group of NADP were modeled as glutamate molecules in PRCM of the initial stage (Fig. 6A, Supplementary Note 3, Tables S3‒S4 and Figs. S16‒S17), and in PRCM-2 and PRCM-3 of the steady stage (Fig. 6D).

## Discussion

For GDH in action, we identified four conformations in the initial stage and seven in the steady stage using cryoEM (Fig. 3). The conformations were in variety with respect to the NAD-domain motion and the association modes of the cofactor and ligand molecules (Figs. 3, 4, 5, 6, Supplementary Notes 2‒3 and Figs. S4–S18). After speculating which of NADP or NADPH was bound to the active-site cleft of each NAD-domain conformation, we discuss the stage-dependent pathways and dynamics during the cofactor-ligand association and catalysis.

Using spectroscopy or crystallography, previous studies reported relationship between reduction/oxidization of cofactor molecules bound to enzymes and order/disorder of their ribose-nicotinamide groups^35–40^. A spectroscopic study on dihydrofolate reductase revealed that NADPH was more rigid than NADP due to the *cis*- and *trans*-conformational changes and the instability in the interaction of the oxidized nicotinamide ring with the enzyme^35^. The reduced form of nicotinamide adenine dinucleotide (NADH) bound to aldehyde dehydrogenase retains the mobility, but the extent of the motion is less than that of the oxidized state (NAD)^36^. In crystal structure analyses^37–39^, the conformational disorder of the ribose-nicotinamide group was prominent in NADPH rather than NADP, for instance, in aldehyde dehydrogenase-cofactor complex. Therefore, conformational disorder of the ribose-nicotinamide group may depend on the interactions in the binding pockets. Furthermore, the order/disorder was under the influence of the ionic strength^40^.

In this study, as GDH was dissolved in a solution at a low ionic strength and neutral pH, we first assumed that the ribose-nicotinamide group was rigid in NADPH and flexible in NADP according to the results from the spectroscopic studies^35,36^. Therefore, NADPH was modeled for the maps in PROP, HLOP-α1, and HLOP-β, in which the entire structures of cofactor molecules were resolved (Fig. 5, Supplementary Note 3 and Table S3). In the case of HLOP, HLOP-α2, HLOP-γ, and PRCM, the maps of cofactor molecules were interpreted as the adenosine-pyrophosphate group of NADP with disorder at the ribose-nicotinamide groups (Fig. 6, Supplementary Note 3, Tables S3‒S4 and Figs. S16‒S17).

**Figure 7:**
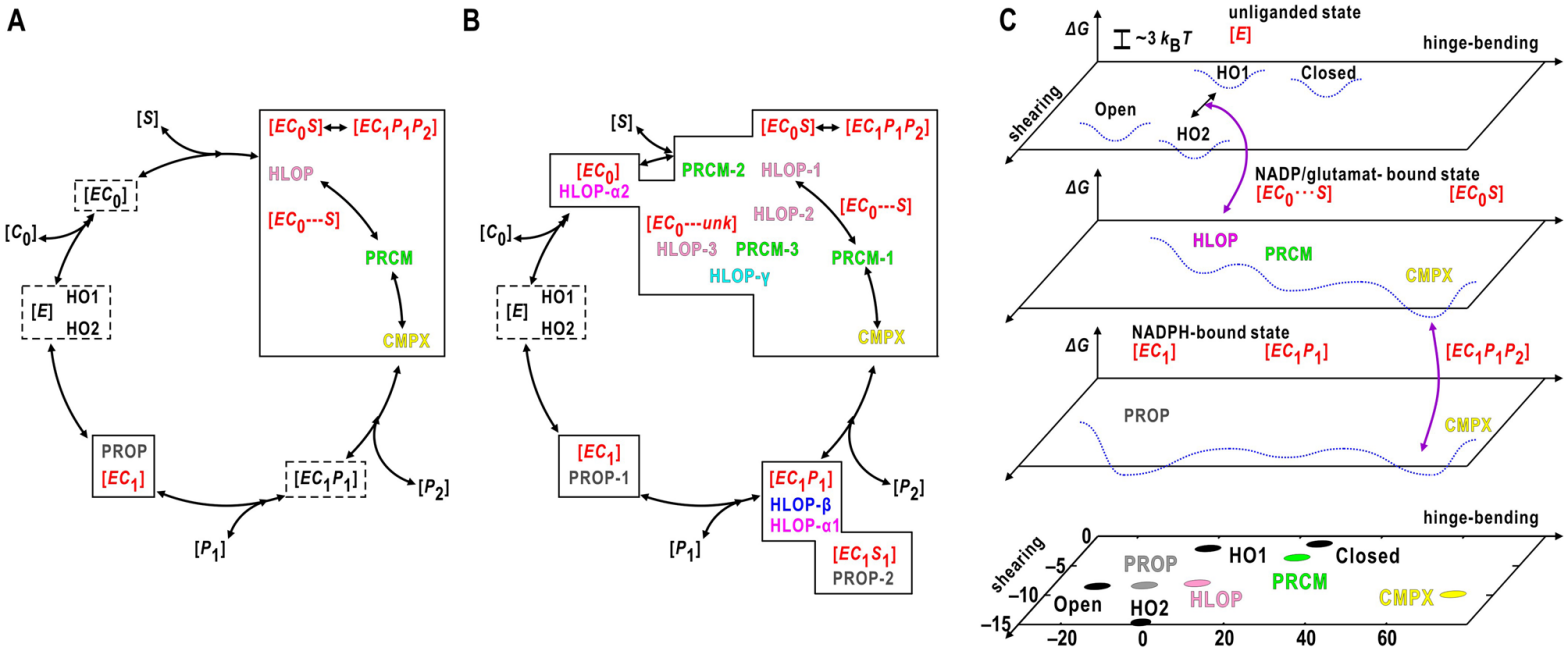
Schematic diagram on the enzymatic reaction from the viewpoint of conformation. Hypothetical reaction cycles for the initial (**A**) and steady (**B**) stages, under assuming that the nicotinamide group is more rigid in NADPH than NADP. The observed states (colored in red) in Fig. 1D are indicated by solid boxes, while missed states by dashed boxes. The observed conformations are shown in the coloring scheme in Fig. 3D. (**C**) Schematic on energy landscape and cofactor/ligand binding in the initial stage of panel (**A**). The first, second, and third panels illustrate the energy landscape (dotted curves) in [*E*], [*EC*_0_···*S*]-[*EC*_0_*S*], and [*EC*_1_*P*_1_*P*_2_]-[*EC*_1_*P*_1_]-[*EC*_1_], respectively, on the plane spanned by vectors representing the hinge-bending and shearing motions of the NAD domain. The bottom panel is taken from Fig. 3D for the initial stage.

For the initial stage, we propose a hypothetical reaction pathway in the deamination reaction composed of four metastable conformations (Fig. 7A). HLOP had a similar NAD-domain conformation to non-productive GDH-NADP complex^25^, and probably appears during the shearing motion between HO1 and HO2 of the unliganded state (Fig. 3D) to act as a platform for cofactor and ligand binding (Fig. 6B). Therefore, HLOP with the incompletely closed active-site cleft is designated as [*EC*_0_···*S*] state. In the state, although cofactor and substrate molecules are bound to the active-site cleft, the reaction is impossible in the incompletely closed cleft.

We missed maps of GDH-glutamate complex with a closed NAD-domain conformation as observed in *C. symbiosum* GDH-glutamate complex^34^. As the NAD-domain conformation probably prevents the access of cofactor molecule to the binding site, NADP molecule bound to HLOP may associate with the active-site cleft prior to the access of glutamate. The scenario of the ordered bi reaction assumes that NADP molecule facilitates the penetration of glutamate molecules into the ligand-binding pocket, for instance, through the binding site flanking the pyrophosphate group (Fig. 6A,D). One of the Arg187 sidechain conformers pointing toward the ligand-binding pocket may assist the access of glutamate molecule to the pocket (Supplementary Fig. S14). On the other hand, the conformational fluctuation of the nicotinamide group in the bound NADP molecule (Fig. 6B) may prevent the closing motion from HLOP to PRCM.

PRCM with the incompletely closed active-site cleft was also assigned as [*EC*_0_···*S*]. NAD-domain motion to spontaneously and/or stochastically close the active-site cleft transforms HLOP to PRCM within the [*EC*_0_···*S*] state. The size of the active-site cleft of PRCM slightly narrower than that of HLOP likely prevents further penetration of cofactor and ligand molecules into the cleft, but the conformational disorder of the nicotinamide group (Fig. 6D) probably still hinders the closing motion from PRCM to CMPX.

Once the nicotinamide group stochastically adopts conformations suitable for closing the active-site cleft, PRCM is transformed to CMPX. Then, the position of the bound glutamate molecule will be finely tuned suitable for the reaction by the support of the sidechains of Arg187 and the other residues forming the pocket (Fig. 4E‒G). The absence of conformations in between PRCM and CMPX (Fig. 3D) implies that the PRCM-CMPX transformation occurs quickly and/or through other conformations unresolvable in this study.

Once CMPX as [*EC*_0_*S*] is formed, CMPX catalyzes the reaction and becomes [*EC*_1_*P*_1_*P*_2_] (Fig. 4A‒D). Then, the sidechains forming the ligand-binding pocket were almost the same conformations between [*EC*_0_*S*] and [*EC*_1_*P*_1_*P*_2_] to fix the ligand molecules for efficient reactions (Fig. 4F,G). The large population of CMPX in the initial stage (Fig. 3C) may be explained by the low probability of transformation from CMPX to PRCM and from CMPX to PROP.

After the conversion of [*EC*_0_*S*] to [*EC*_1_*P*_1_*P*_2_] in CMPX, the NAD-domain motion opens the active-site cleft and support the escape of ammonium ion and 2-oxoglutarate molecule from the ligand-binding pocket (Fig. 5A). As [*EC*_1_*P*_1_] was missed in the initial stage, the two product molecules quickly escaped from the pocket. One of the two conformers of the Trp89 sidechain prevents the closing motion of the NAD domain (Supplementary Fig. S14) and may allow the penetration of hydration water molecules into the pocket to produce [*EC*_1_] (Fig. 5A). As maps corresponding to [*E*] were missed, GDH may immediately associate with NADP or NADPH after the release of NADPH from [*EC*_1_].

This hypothetical pathway for the initial stage suggests that the Michaelis complex is an ensemble of heterogeneous conformations, HLOP, PRCM and CMPX. The free-energy barriers among the three may be in a level of a few *k*_B_*T*, where *k*_B_ is the Boltzmann’s constant and *T* is the absolute temperature, as in revealed for the NAD-domain conformations in unliganded state^21^. Then, HLOP, PRCM, and CMPX may be reversibly transformed from each other by the NAD-domain motion and the conformational fluctuation of the nicotinamide group of NADP molecule. In this regard, a series of spectroscopic studies on lactate dehydrogenase demonstrated that the Michaelis complex of the enzyme is an ensemble of heterogeneous with respect to the electronic and vibrational states^2,3^. Therefore, the present study suggests that heterogeneity of the Michaelis complex extends over whole molecule.

In the steady stage, the main pathway is basically the same as that in the initial stage. However, the three additional NAD-conformations (HLOP-α, HLOP-β, and HLOP-γ) and derivatives of PROP, HLOP and PRCM probably induce bifurcations in the pathway (Fig. 7B). Subsequently, the reaction velocity will be reduced from that of the initial stage, and the assumption made by the kinetic theory for the initial stage is broken down.

HLOP-1 acts as an NADP-binding platform (Fig. 6B and Supplementary Fig. S17C), while HLOP-2 and HLOP-3, in which the adenosine-pyrophosphate groups of the NADP molecules are weakly associated with the NAD domains, may reflect structures at the instance of the association/dissociation of NADP molecules. The similarity of maps in the ligand-binding pocket suggested that HLOP-α2 and HLOP-γ are likely produced by the opening and closing motions of the NAD domain from HLOP-3, respectively, along with the exchange of a glutamate molecule and two hydration water molecules (Supplementary Fig. S17D). PRCM in the initial stage likely splits into PRCM-1 and PRCM-2. PRCM-1 may be a transient conformation from HLOP-1 and HLOP-2 to CMPX. PRCM-3 may be interpreted as a closed form of HLOP-3 (Fig. 6B,D). In the three PRCM subclasses, as the Arg187 sidechains were moved out from the ligand-binding pocket (Supplementary Fig. S15), the closing motion from PRCM to CMPX will bring again the sidechains into the pocket.

PROP-1, PROP-2, HLOP-α1, and HLOP-β may be derivatives of PROP in the initial stage with the variation of the molecules in the ligand-binding pocket (Fig. 5B‒E). Although PROP with two hydration water molecules was major in the initial stage, the penetration of 2-oxoglutalate or glutamate molecules into the pocket may facilitate the slight opening of the active-site pocket from PROP in concert with the conformational variety in the Trp89 sidechains (Supplementary Fig. S15).

Other reaction cycles may be possible depending on the interpretation for the maps of cofactor and ligand molecules. For instance, when we interpret the order/disorder of cofactor maps according to the crystal structure analyses and ignore the map shapes and the correlation coefficients and *Q*-scores, an alternative hypothetical cycle is possible (Supplementary Fig. S19). In the initial stage, PROP acts as [*EC*_0_]. As we missed conformations assignable as [*EC*_0_···*S*] between PROP and CMPX, substrate-binding and large NAD-domain motion occur very quickly and/or through other conformations unresolved in the present study. As cofactors in HLOP and PRCM are interpreted as NADPH, they are assigned as [*EC*_1_*P*_1_] or [*EC*_1_*P*_1_*P*_2_] with the opened active-site cleft. In the steady stage, the accumulation of the derivatives from HLOP and PRCM may hinder the production of [*E*] or [*EC*_1_] state.

In the selected classification trials, 24% of GDH images in the initial stage and 36% in the steady stage were discarded due to blurred NAD-domain maps and an insufficient number of images (Fig. 3C). However, as the core domains can be modeled even in the discarded subclasses, the discarded images may be minor NAD-domain conformations that are too few to identify the NAD domain in the present classification. Therefore, a large number of images may be advantageous to resolve minor and/or unstable conformations and reconstruct maps with higher resolution to distinguish the reduced/oxidized state of cofactor molecule and the ligand molecules in the pocket. In addition, in the classification of the NAD-domain conformations, we can use the manifold learning without any assumption on the number of conformations. The manifold learning is powerful for illustrating detailed pathways, as demonstrated for complicated systems, such as conformational changes in adenylate kinase^41^ and the growth process of mesoscale particles^42^.

Hydration water molecules exert a great influence on protein motion as reported^21,23–25^. For instance, a hydration water molecule that hinders the closing motion of the NAD domain was identified on the sidechain of Trp89 in PRCM-3 (Supplementary Fig. S15) as predicted in our simulation study^24^. Therefore, simultaneous visualization of metastable conformations and hydration structures is necessary. Although hydration water molecules were identified inside the core domains, the number of hydration water molecules is still smaller than that in the crystal structures. Toward understanding the correlation between stochastic hydration changes and domain motion^24^, the use of the prediction methods for hydration structures helps to identify hydration sites in potential maps^43^.

During the action, GDH associates with a NADP molecule in the first step and a glutamate molecule in second step. As the cofactor- and ligand-binding alters the internal energy of the complex from that in the unliganded state, visualization of the energy landscape over the cycle is probably difficult. Instead, when we separately focus on pairs of conformations, such as HLOP and PRCM, that are adjacent in the hypothetical pathway and associate with the same cofactor and ligand molecules (Fig. 7C), the energy landscape may be visualized using our theory applied to the NAD-domain motion in the unliganded state^21^. In addition, to estimate the changes in the energy landscape before and after cofactor and ligand association, the parallel cascaded selection MD^44^ under a set of appropriate force-field parameters^31,45^ may explore pathways of conformational changes between different complexes.

## Methods

### Preparation of GDH

GDH from *T. profundus*^46^ was expressed in an *Escherichia coli* DB21 strain transformed with a pET-26b expression vector carrying the wild-type GDH gene. A crude GDH solution was obtained by 10-min heat treatment at 353 K for a freeze-and-thawed culture solution. GDH was purified using HiTrap Q HP anion-exchange, HiTrap Phenyl HP hydrophobic-interaction, RESOURCE PHE hydrophobic-interaction, and RESOURCE Q anion-exchange columns (GE Healthcare, Chicago, IL, USA). The elution from the RESOURCE Q anion-exchange column was demineralized and purified further by affinity chromatography using Reactive Red 120 gel (Sigma-Aldrich, St. Louis, MO, USA). The purity of the GDH solution was examined by sodium dodecyl sulfate–polyacrylamide gel electrophoresis after each chromatography. The purified GDH solution was concentrated using a Centriprep YM50 tube (Merck Millipore, Burlington, MA, USA). Approximately 10 mg of GDH was obtained using the purification protocol for a 1-L culture.

### Enzymatic activity measurement and analysis

The enzymatic activity of GDH at 293 K was measured for a solution containing 24 μg/mL (86 nM of GDH hexamer or 516 nM of subunit) GDH, 0.5–4.0 mM sodium glutamate (Wako, Osaka, Japan), 0.1–1.0 mM NADP (Nakarai tesque, Kyoto, Japan), and 50 mM Tris (hydroxymethyl amino methane) (Wako, Osaka, Japan). The pH of each solution was 7.5. As NADPH produced in the reaction exhibits an absorption peak at 340 nm with a molar extinction coefficient of 6,200 mM^−1^ cm^−1^, the progress of the enzymatic reaction was monitored by changes in the absorption at 340 nm using a UV-recording spectrophotometer U-2900 (Hitachi-Hi-Tech Science, Tokyo, Japan). The measurement was started 5-s after mixing the GDH, glutamate, and NADP solutions. The initial velocity of the reaction was calculated from data within the first 10% of the extent of the reaction achieved at the steady stage.

The L‒B plot was obtained from the enzymatic reaction data measured at five glutamate and four NADP concentrations (Fig. 1E). Assuming an ordered bi reaction (Fig. 1D), the data points obtained at an NADP concentration are approximated by the following equation:

$$\frac{1 }{{v}_{0 }}=\frac{{K}_{{\text{m}}}^{{\text{Glu}}}}{{V}_{{\text{max}}}}\left(1+\frac{{K}_{{\text{d}}}^{{\text{NADP}}}}{{\left[{\text{NADP}}\right]}_{0}}\right)\frac{1}{{\left[{\text{Glu}}\right]}_{0}}+\frac{1}{{V}_{{\text{max}}}}\left(1+\frac{{K}_{{\text{m}}}^{{\text{NADP}}}}{{\left[{\text{NADP}}\right]}_{0}}\right)$$

where ${v}_{0}$ denotes the initial velocity. ${V}_{max}$ is the maximum velocity. ${K}_{m}^{\text{NADP}}$ and ${K}_{d}^{\text{NADP}}$ are the Michaelis constant and dissociation constant of NADP, respectively. ${K}_{m}^{\text{Glu}}$ is the Michaelis constant of glutamate. ${\left[{\text{NADP}}\right]}_{0}$ is the initial NADP concentration. The intercept and gradient values of the regression lines in the L‒B plot linearly depended upon the reciprocal of ${\left[{\text{NADP}}\right]}_{0}$ (inset of Fig. 1D). The parameters governing the kinetics were determined and are listed in Supplementary Table S1.

### CryoEM

The purified GDH solution was mixed with NADP and glutamate solution prior to flash-freezing. The mixed solution contained 17.1 µM GDH (corresponding to 4.8 mg mL^−1^), 0.5 mM NADP, 100 mM Glutamate, and 5 mM Tris–HCl. The pH of the mixed solution was 7.5 as confirmed before the preparation and, the stoichiometry of NADP and glutamate molecules against a single GDH subunit was approximately 5 and 975, respectively. In addition, the concentration of glutamate was comparable to that in vivo^47^, while that of NADP was 250-fold higher.

In the initial stage, as the rapid reaction at 297 K made it difficult to perform the flash-freezing procedure, the solution maintained at 277 K was flash-frozen 15-s after mixing the GDH, NADP, and glutamate solutions. For the steady stage, the specimen solution was flash-frozen 1-h after the mixing at 293 K.

An aliquot of 3-µL specimen solution was placed on a glow-discharged Cu R1.2/1.3 200-mesh holey carbon grid (Quantifoil, Großlöbichau, Germany). After removing an excess amount of the specimen solution under 100% relative humidity at 281 K, the grid was flash-frozen by liquid ethane using a Vitrobot device (Thermo Fisher Scientific, Waltham, MA, USA).

CryoEM observations of the frozen-hydrated specimens in the initial and steady stages were performed using a CRYO ARM300 system (JEM-Z300FSC; JEOL, Tokyo, Japan) operated at an acceleration energy of 300 kV. The system was equipped with an Ω-type energy filter and allows us to record zero-energy-loss (10 eV) images. We used a Gatan K3-summit detector (Gatan, Pleasanton, CA, USA) operated by the counting mode and at a calibrated magnification of 60,000 that yielded a pixel size of 0.752 Å. CryoEM images were automatically collected at 77 K using *SerialEM* data acquisition software^48^. The 2-s exposure was fractionated into 50 frames, and the irradiation dose was 1.0 e^−^/Å^2^ per frame. The defocus values were in the range of 0.4–4.8 μm.

### Image processing

We obtained 7,750 and 7,075 micrographs of the initial and steady stages, respectively. First, dose-fractionating motion-correction was applied to the micrographs using *MOTIONCOR2*^49^. Next, we estimated the parameters of the contrast transfer function (CTF) using *CTFFIND 4.1*^50^.

Subsequent analyses of the initial and steady stages were performed using *Relion 4* and *Relion 4β*^51^. The auto-picking procedure for the initial stage was performed using a TOPAZ neural network^52^ that was retrained using approximately 100,000 GDH particles selected from picked particles. The obtained particle images were subjected to reference-free two-dimensional (2D) classification followed by three-dimensional (3D) classification referring to a 3D model constructed by de novo 3D model generation^53^. The two classification processes eliminated aggregates of GDH molecules and irrelevant images, such as ice contamination and mis-picked edges of carbon holes, and selected 430,904 and 423,327 good-quality images for the initial and steady stages, respectively.

The selected images were then used in the 3D-refinement procedures^54^ imposing D3 symmetry with an angular sampling of 1.8 degrees. After the process, the CTF parameters for each image were refined. Subsequently, Bayesian polishing^55^ was applied to only the first 20 frames of each image, which had estimated nominal B-factors greater than – 40 Å^2^. This procedure was effective in avoiding the influence of the latter frames that exhibited severe radiation damage and ensuring a sufficient signal-to-noise ratio for each image to accurately determine the particle orientation. Polished images were used in the final 3D auto-refinement procedure imposing D3 symmetry with an angular sampling of 0.9 degrees.

The potential map was then sharpened by applying a negative B-factor estimated from the power spectrum of the potential map^56^. The *blocres* program implemented in *Bsoft* was used to estimate the variations in local resolution^57,58^. The resolution of the final map (Fig. 2) was estimated based on the GS-FSC criterion of 0.143^56^ (Supplementary Figs. S1C and S2C). In this study, all reported resolutions were based on gold-standard refinement procedures and the corresponding solvent-flattened FSC with a criterion of 0.143^56^. The FSC curve was then corrected for the effects of a soft mask using high-resolution noise substitution^59^.

### Focused classification on the NAD-domain conformations

We performed a two-step focused classification to separate the conformational variations of both the NAD-domain conformations and cofactor/ligand modes (Supplementary Fig. S4). First, we applied the symmetry expansion method^13^ to each image for generating five replicas related to the D3 symmetry. Then, a mask for the NAD domain together with a part of the core domain and the active-site cleft was constructed by referring to the subunit structures observed in the previously obtained MD trajectory^24,31^. We subtracted the other parts that were composed of the five core domains and NAD domains from six symmetry-expanded particle images.

Focused 3D classification was performed for a fixed orientation of the NAD domains. The NAD-domain images were then separated into a given number of classes. For each class, 3D auto-refinement was applied to the original images to avoid artifacts originating from inaccurately prepared signal-subtracted images of the other NAD domains.

Even after the classification, the maps of NADP molecules were insufficient to determine the orientation and conformations of NADP molecules. Therefore, we applied the second classification to separate each of the classes into subclasses using the procedure in the first classification described above.

As the focused classification applied to noisy and low contrast images does not always converge to a unique set of maps as pointed out^28^, we conducted independently several classification trials using *Relion* and *cryoSPARC*^60^ (Supplementary Note 2 and Figs. S5‒S10). We validated whether the sets of classified subclass maps were consistent among different classifications by inspecting the maps and calculating the correlation coefficient between any pair of maps.

### Structure model for refined maps from a focused classification

After the focused classification trials, structural models were constructed for the map in selected trial using *Coot*^61^ (Supplementary Table S2 and Figs. S11‒S12). Then, we used the crystal structure model of the unliganded state refined at a resolution of 1.8 Å (the PDB accession code: 1EUZ)^21^. The cofactor and ligand molecules were manually modeled by inspecting the potential map, and hydration water molecules were introduced with reference to the crystal structure model. Each model was refined in real space using *Phenix*^62,63^, and the conformations and stereochemistry were manually corrected according to a report from the PDB validation server. For cofactor, ligand and solvent molecules in the refined structure models, we examined how well the atomic models reproduce the maps using the correlation coefficient^32^, and measured the resolvability of their maps using the *Q*-scores^33^ (Supplementary Note 3, Tables S3‒S4 and Figs. S16‒S17).

### Principal component analysis

To quantitatively describe the NAD-domain motions, the representative models built for the subclass maps were projected onto the plane defined by the two principal component vectors that were deduced from the principal component analysis (PCA)^64^ for representative 71 structures obtained in our previous MD simulation^31^. Prior to the PCA for MD structures, each representative MD model was superimposed to a reference with respect to a set of Cα-atoms in rigid secondary structures of the core domain was used as a reference to superimpose subunits. Then, PCA for the NAD-domain conformations was carried out using the Cα-atoms in the Rossmann fold region of the NAD domain. The obtained first and second principal component (PC) vectors described the hinge-bending and shearing motions of the NAD domains, respectively. The contribution of the two motions to the variety of the NAD conformations was approximately 93%, as indicated in the inset of Fig. 3D.

Each refined structure from the initial and steady stages was projected onto the two axes to quantitatively measure the magnitudes of the hinge-bending and the shearing motions. In addition, we also projected structural models obtained in the previous studies as references. They were cryoEM models for the unliganded state^22^ and the GDH-NADP complex^26^, and the crystal structure model for the unliganded wild-type^21^ GDH (Fig. 3D).

### Supplementary Information

Supplementary Information.

## Data Availability

The datasets used and/or analyzed during the current study available from the corresponding author on reasonable request.

## Code availability

Representative EM maps in the initial stage were deposited to EMDB as the accession codes EMD-38249 (PROP), EMD-38250 (HLOP), EMD-38251 (PRCM), EMD-38252 (CMPX-A) and EMD-38253 (CMPX-B). Those in the steady stage were EMD-38254 (PROP-1), EMD-38255 (PROP-2), EMD-38256 (HLOP-α1), EMD-38257 (HLOP-α2), EMD-38261 (HLOP-β), EMD-38262 (HLOP-γ), EMD-38258 (HLOP-1), EMD-38259 (HLOP-2), EMD-38260 (HLOP-3), EMD-38263 (PRCM-1), EMD-38264 (PRCM-2), EMD-38265 (PRCM-3), EMD-38266 (CMPX-1) and EMD-38267 (CMPX-2). In addition, the structural models built for those maps were deposited to the PDB as accession codes 8XCO (PROP), 8XCP (HLOP), 8XCQ (PRCM), 8XCR (CMPX-A) and 8XCS (CMPX-B) in the initial stage, and 8XCT (PROP-1), 8XCU (PROP-2), 8XCV (HLOP-α1), 8XCW (HLOP-α2), 8XD0 (HLOP-β), 8XD1 (HLOP-γ), 8XCX (HLOP-1), 8XCY (HLOP-2), 8XCZ (HLOP-3), 8XD2 (PRCM-1), 8XD3 (PRCM-2), 8XD4 (PRCM-3), 8XD5 (CMPX-1) and 8XD6 (CMPX-2) in the steady stage.
