# Supplementary material for: CryoEM-sampling of metastable conformations appearing in cofactor-ligand association and catalysis of glutamate dehydrogenase
Source: Sci Rep. 2024 May 15;14:11165. doi: 10.1038/s41598-024-61793-x (PMC11096400; doi:10.1038/s41598-024-61793-x)
Supplement: Supplementary file 1 — Supplementary Information. [file 41598_2024_61793_MOESM1_ESM.docx]

**Supplementary Information**

CryoEM-sampling of metastable conformations appearing in cofactor-ligand association and catalysis of glutamate dehydrogenase

Taiki Wakabayashi^1,2^, Mao Oide^1,2,3,4^ and Masayoshi Nakasako^1,2^

^1^Department of Physics, Faculty of Science and Technology, Keio University, 3-14-1 Hiyoshi, Kohoko-ku, Yokohama, Kanagawa 223-8522, Japan.

^2^RIKEN SPring-8 Center, 1-1-1 Kohto, Sayo-cho, Sayo-gun, Hyogo 679-5148, Japan.

^3^PRESTO, Japan Science and Technology Agency, Chiyoda-ku, Tokyo, 102-0076, Japan

^4^Protein Research Institute, Osaka University, Yamadaoka, Suita, Osaka, 565-0871, Japan

Corresponding author: Masayoshi Nakasako

**Supplementary Table S1**

**Kinetic parameters of the ordered bi reaction of GDH**

| $V_{\max}$ (μM s^–1^) | 1.45 ± 0.1 |
| --- | --- |
| $K_{\text{m}}^{\text{Glu}}$ (μM) | 2380 ± 24 |
| $k_{\text{cat}}$ (s^–1^)^*^ | 2.78 ± 0.00 |
| $K_{\text{m}}^{\text{NADP}}$ (μM) | 176 ± 2 |
| $K_{\text{d}}^{\text{NADP}}$ (μM) | 46.2 ± 0.4 |

$V_{max}$ is the maximum velocity. $K_{m}^{\text{Glu}}$ is the Michaelis constant of glutamate. The catalytic constant of the reaction, $k_{\text{cat}}$, was calculated as $V_{\max}/\left[\text{GDH}\right]_{0}$, where $\left[\text{GDH}\right]_{0}$ is the initial concentration of GDH subunits. $K_{m}^{\text{NADP}}$ and $K_{d}^{\text{NADP}}$ are the Michaelis constant and the dissociation constant of NADP, respectively.


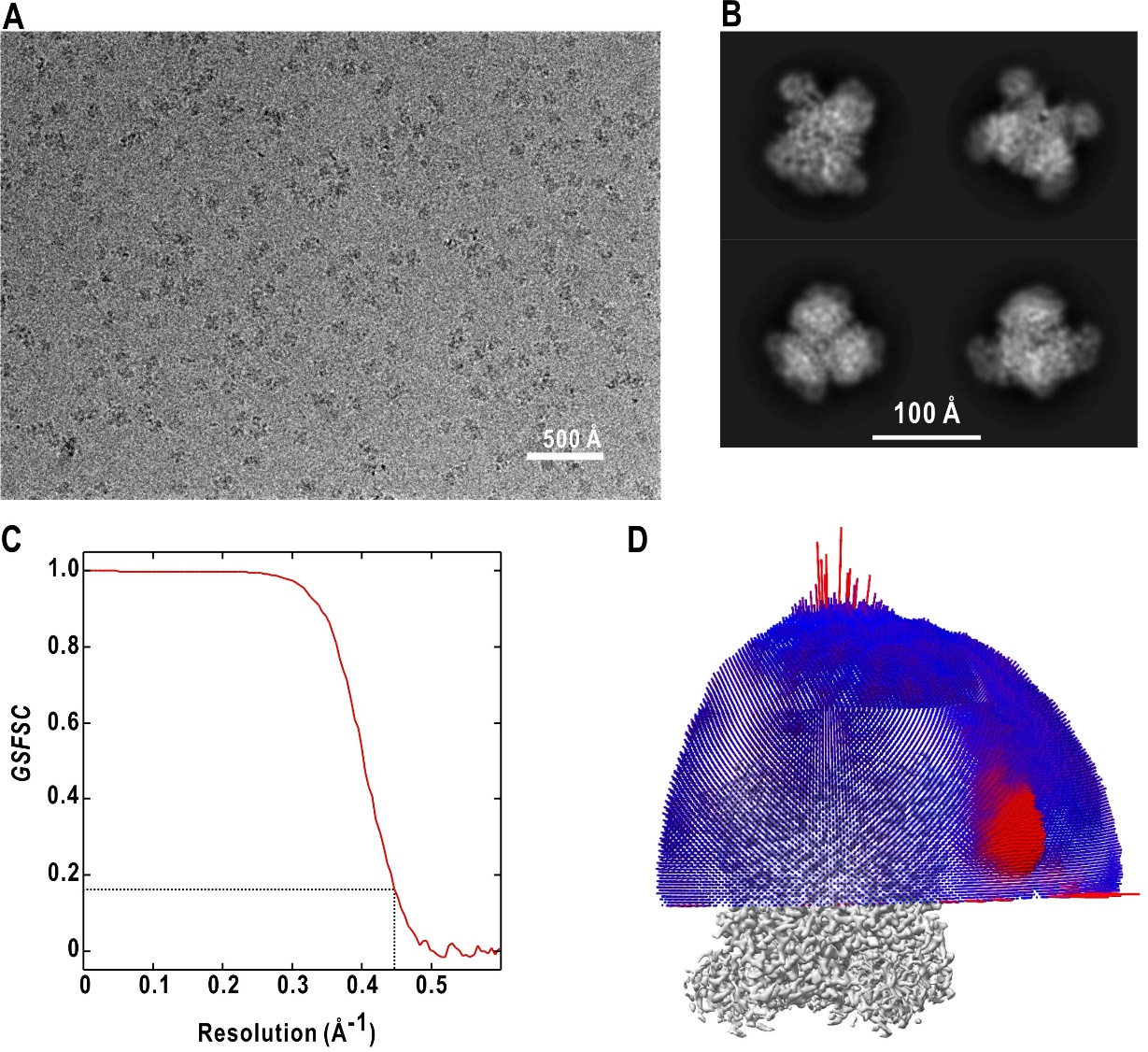


**Supplementary Figure S1**

**Structure analysis for the initial stage of the enzymatic reaction.**

(A) Electron micrograph of frozen hydrated GDH molecules in the initial stage. (B) Examples of averaged molecular images of GDH in four classes after 2D classification. (C) Gold-standard Fourier shell correlation (*GSFSC*) curve for the reconstructed potential map in Fig. 2A of the main text. The effective resolution of the map was determined from the spatial frequency at which the FSC value dropped to 0.143^1^. (D) Eulerian plot showing the frequencies regarding the orientation of GDH molecules against the direction of the incident electron beams in EM images. Panel (D) was prepared using *UCSF ChimeraX*^2^.

**References**

1. Rosenthal, P. B. & Henderson, R. Optimal determination of particle orientation, absolute hand, and contrast loss in single-particle electron microscopy. *J. Mol. Biol.* **333**, 721–745 (2003).
2. Pettersen, E. F., Goddard, T. D., Huang, C. C., Meng, E. C., Couch, G. S., Croll, T. I., Morris, J. H. & Ferrin, T. E. *UCSF ChimeraX*: Structure visualization for researchers, educators, and developers. *Protein Sci*. **30**, 70–82 (2021).

**
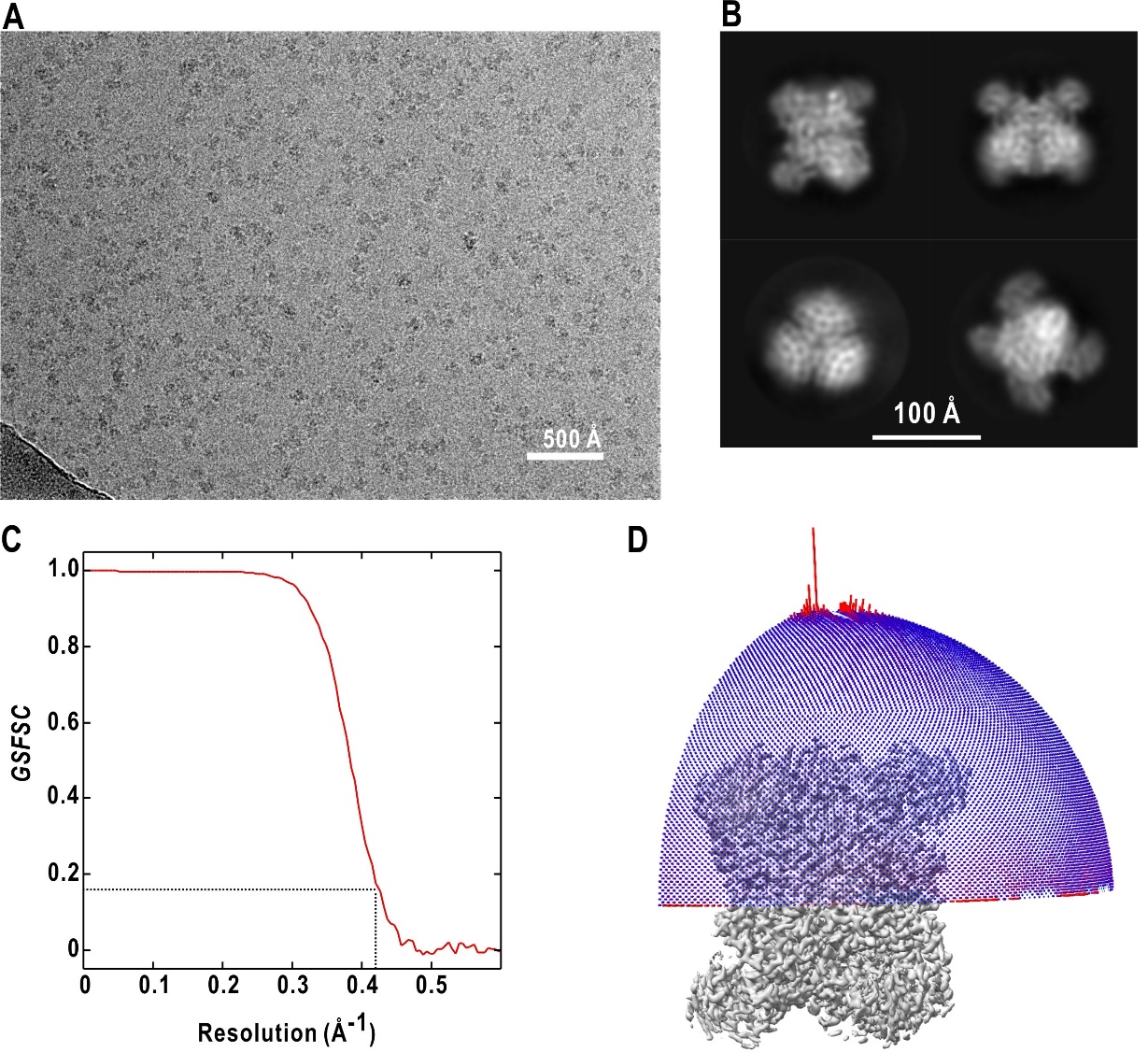
**

**Supplementary Figure S2**

**Structure analysis for the steady stage of the enzymatic reaction.**

(A) Electron micrograph of frozen hydrated GDH molecules in the steady stage. (B) Examples of averaged molecular images of GDH in four classes after 2D classification. (C) Gold-standard Fourier shell correlation (*GSFSC*) curve for the reconstructed potential map in Fig. 2B of the main text. The effective resolution of the map was determined from the spatial frequency at which the FSC value dropped to 0.143^1^. (D) Eulerian plot showing the frequencies regarding the orientation of GDH molecules against the direction of the incident electron beams in EM images. Panel (D) was prepared using *UCSF ChimeraX*^2^.

**References**

1. Rosenthal, P. B. & Henderson, R. Optimal determination of particle orientation, absolute hand, and contrast loss in single-particle electron microscopy. *J. Mol. Biol.* **333**, 721–745 (2003).
2. Pettersen, E. F., Goddard, T. D., Huang, C. C., Meng, E. C., Couch, G. S., Croll, T. I., Morris, J. H. & Ferrin, T. E. *UCSF ChimeraX*: Structure visualization for researchers, educators, and developers. *Protein Sci*. **30**, 70–82 (2021).

**Supplementary Note 1**

**Small-angle X-ray scattering**

The D3 map of the GDH hexamer in the steady stage was larger than that in the initial stage and slightly smaller than that in the unliganded state. To examine whether conformational changes were induced by the flash-cooling, we analyzed the solution structure of GDH in the steady state using small-angle X-ray scattering (SAXS). As SAXS comes from an ensemble average of the structure factors of possible protein conformations in solution at ambient temperature, SAXS is advantageous to examine whether flash-cooling has influences to the overall structures of proteins in solution. Although we were also interested in the SAXS of the initial stage, currently the measurement was still difficult without a fast-mixing chamber.

*SAXS experiment*

SAXS measurements were carried out at BL38B2 of SPring-8. The wavelength of the X-ray was tuned to 1.0000 Å, and the camera distance was 2,500 mm. The temperature of the specimen solution was maintained at 293 K. SAXS patterns were recorded as a series of 10 frames of 3 s exposure using a PILATUS 300K-W detector (Dectris, Baden-Daettwil, Switzerland).

GDH in the unliganded state was dissolved in 5 mM Tris-HCl buffer. For the steady stage, GDH was dissolved in 0.5 mM NADP, 100 mM sodium glutamate and 5 mM Tris-HCl solution. The pH of each specimen solution was adjusted at 7.5. We measured a series of GDH concentrations at 1, 2, and 3 mg/mL using a single specimen cell with quartz windows of 10 μm thickness and a path length of 3.0 mm. The SAXS pattern from the buffer solution was used as the background for the GDH solution.

*SAXS data processing and analysis*

The two-dimensionally recorded SAXS pattern of each frame was radially averaged to a one-dimensional profile. The profile of the buffer solution was then subtracted from that of the GDH solution, and then a net profile of the GDH was obtained by accumulating profiles over the frames after the normalization with respect to the volume and concentration.

The Guinier approximation^1^ was applied to the profiles in a small-angle region: the scattering intensity *I*(*S*,*C*) from a diluted specimen solution of concentration *C* at a scattering vector length *S* is approximated using the zero-angle scattering intensity *I*(*S*=0,*C*) and the radius of gyration *R*g(*C*) as:

$$I\left(S,C\right)=I\left(S=0,C\right)\text{ exp}\left[-\frac{4\pi^{2}}{3}R\text{g}\left(C\right)^{2}\text{ }S^{2}\right] ,\text{ }S=\frac{2\sin\theta}{\lambda}$$

where 2*θ* is the scattering angle, and *λ* is the X-ray wavelength. For a dilute solution, *I*(*S*=0, *C*) and *R*g(*C*)^2^ approximately depend on *C* as^1^:

$\frac{KC}{I\left(S=0,C\right)}=\frac{1}{M_{\text{w}}}+2A\cdot C,\text{ }R\text{g}\left(C\right)^{2}=R\text{g}\left(C=0\right)^{2}-B\cdot C$,

where *K* denotes an experimental constant. *M*_w_ is the apparent molecular weight of the protein. *A* and *B* are the second virial coefficient and the parameter reflecting the mode of intermolecular interactions, respectively^1^. The distance distribution function *P*(*r*) was calculated using the program *GNOM*^2^.

*SAXS changes between the unliganded state and the steady stage*

Each SAXS profile of GDH (Fig. S3A) was approximated by a Gaussian in S < 0.008 Å^–1^, as demonstrated by the Guinier plots (Fig. S3B). Little GDH molecules formed non-specific aggregates. We determined *I*(*S*=0, *C*) and *R*g(*C*) values at three different GDH concentrations using the Guinier approximation. Based on the concentration dependence (Fig. S3C), the *R*g(*C*=0) values in the unliganded state and in the steady stage were determined to be 43.2 ± 0.1 Å and 42.2 ± 0.1 Å, respectively. This result implied that the molecular size of the GDH hexamer in the steady stage was slightly smaller than that in the unliganded state. In contrast, the 1/*I*(*S*=0, *C*=0) values were almost constant, because the molecular weight of the GDH hexamer with small cofactor molecules was almost the same as that of the unliganded state.

At *S*=0.017 Å^-1^, an enhancement was characteristic (Fig. S3A) and was explained by the Fourier transform of a cylindrical shape with an approximate diameter and height of 100 nm. The NAD-domain motion in the unliganded state probably blurred the edge of the cylindrical shape of the GDH hexamer, while the NAD-domain motion in the steady stage was suppressed due to interactions between the NAD and core domains mediated by the cofactors. Therefore, the significant increase of intensity at *S*=0.017 Å^-1^ may reflect a more compact structure of the GDH hexamer in the steady stage than that in the unliganded state. In addition, the maximum dimensions estimated from the *P*(*r*) functions in the absence and presence of NADP/glutamate were approximately 120 Å and 115 Å, respectively (the inset of Fig. S3A). These results regarding the size of the hexamer were consistent with the reduction in *R*g(*C*=0) in the presence of NADP/glutamate.

**References**

1. Ginuier, A. & Fournet, G. *Small-Angle Scattering of X-rays.* (Wiley, 1955).
2. Svergun, D. I. Determination of regularization parameter in indirect-transform methods using perceptual criteria. *J. Appl. Cryst.* **25**, 495–503 (1992).


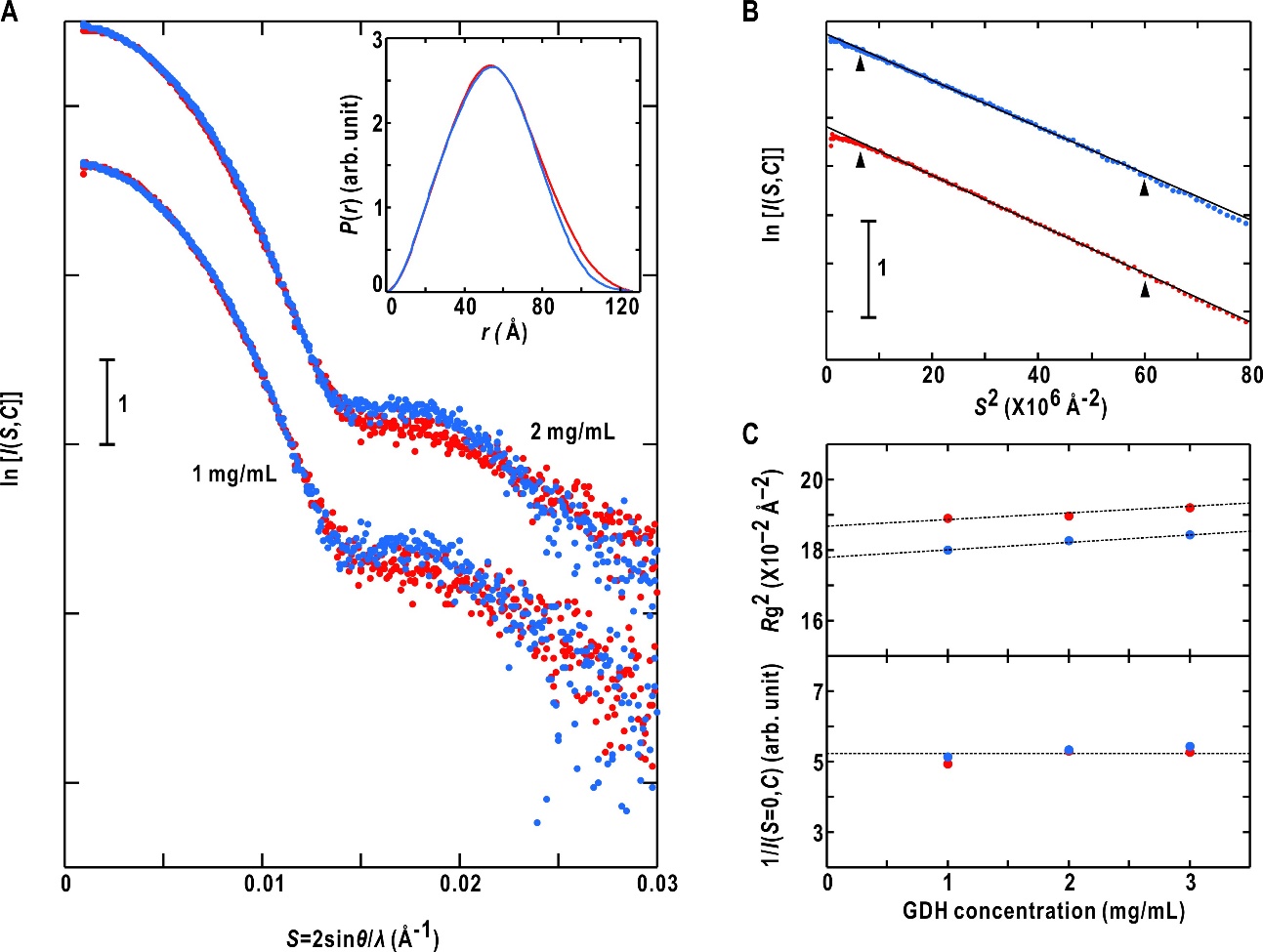


**Supplementary Figure S3**

**SAXS of GDH in the unliganded state and in the steady stage**

(A) SAXS profiles from GDH in the unliganded state (red symbols) and in the steady stage (blue) at GDH concentrations of 1 and 2 mg/mL. The coloring scheme for the unliganded stage and the steady stage is used throughout the panels. For clarity, the profiles at the two concentrations are appropriately shifted. The inset shows the *P*(*r*) functions calculated from profiles from 1 mg/mL solutions. (B) Guinier plots, showing the dependence of the logarithm of the scattering intensity on the square of the scattering-vector length, at a GDH concentration of 1 mg/mL. The two datasets were appropriately shifted for clarity. The least-squares fitting method was applied to the data points between the arrows. The high-resolution edge of the region (*S*^2^ = 60×10^–6^ Å^–2^) satisfied the criteria necessary for the approximation (*SR*g < (2π)^-1^). (C) The concentration-dependences of *R*g(*C*)^2^ (upper panel) and *C*/*I*(*S*=0,*C*) (lower panel). For both the plots, the standard deviation of each data was smaller than the sizes of the symbols.

**Supplementary Note 2**

**Assessment of classification trials**

We applied the focused classification^1^ to the images of GDH to separate the NAD-domain conformations predominantly using *Relion*^2^ (Fig. S4). The focused classification has the potential to separate different conformations from a set of images. Although the orientation of each GDH image against the direction of the incident beam is estimated in the 3D reconstruction, the noise and low-contrast of images may prevent the correct estimation and consequently have influences on the classification of images. Therefore, we conducted independently classification trials, and examined the quality of the classified maps and the correlation scores between maps as described below.

It should be noted that the number of classes assumed in the classification does not necessarily match the number of NAD-domain conformations in the classification for noisy and low-contrast images. Therefore, we estimated the number of NAD-domain conformations by inspecting the classified maps and their similarity.

*Quality of classified maps*

We inspected the classified maps whether the sidechains in the Rossmann fold of the NAD-domain were visible and suitable for model construction (see the panels in the fourth column in Figs. S11 and S12). In addition, we also examined whether the map of adenosine-pyrophosphate groups of a cofactor molecule was well separated from those of residues interacting with the groups (see cofactor maps in Figs. 4-6 in the main text). When a classified maps did not satisfy these conditions, they were categorized to “disordered map”. In contrast, when the maps were suitable for model construction, they were compared with the four representative conformations, CMPX, PRCM, HLOP and PROP in the initial stage (The structural characteristics of the four conformations are described the Results section of the main text).

*Correlation score of maps*

In addition to the visual inspection of the classified maps, we used the following correlation score for a pair of maps to evaluate their similarity:

$score=\left\langle\frac{\left(\psi_{i}\left(\mathbf{r}\right)-\bar{\psi}_{i}\right)\left(\psi_{j}\left(\mathbf{r}\right)-\bar{\psi}_{j}\right)}{\left|\psi_{i}\left(\mathbf{r}\right)-\bar{\psi}_{i}\right|\text{ }\left|\psi_{j}\left(\mathbf{r}\right)-\bar{\psi}_{j}\right|}\right\rangle$,

where $\psi_{i}\left(\mathbf{r}\right)$ is the potential-map value at position $\mathbf{r}$ in the i-th map, and $\bar{\psi}_{i}$ is the average of $\psi_{i}\left(\mathbf{r}\right)$. The score is an average over the NAD-domains. More similar maps of the NAD-domains give higher correlation scores. To calculate the correlation scores, we used *UCSF ChimeraX*^3^ for maps, which were reconstructed from a sufficient number of images, typically more than 50,000, and allowed us the model construction of the NAD-domain, cofactor and ligand molecules.

As references in the subsequent analyses in Figs. S6, S7 and S9, Fig. S5 demonstrates what correlation score is given by a pair of maps. When pairs of maps almost overlap, the correlation score is greater than 0.85. When the correlation score of the maps was 0.80, structural models constructed from the maps differed by less than 1 Å at the top of the NAD-domain. When the correlation score of maps was 0.70, the top of the NAD domain in the constructed models differed by less than 2 Å. Therefore, maps giving the correlation score greater than 0.85 almost overlap, and those giving the score of 0.80 are very similar.

*Correlation scores in classification trials for the initial stage*

For GDH images of the initial stage, we conducted five independent trials by varying the number of classes assumed in the first step (“Trial A”‒“Trial E” in Fig. S6). Fig. S6(A) depicts the correlation scores for the maps within each classification trial (self-correlation). When images of almost the same NAD-domain conformations are separated into different classes, the off-diagonal components in the plots have scores greater than 0.85. Therefore, taking the redundant classification into consideration, we estimated the number of NAD-domain conformations. In the initial stage, four NAD-domain conformations, CMPX, PRCM, HLOP and PROP, were identified in each of the five classification trials. Especially, with respect to the quality of the maps, the “Trial A” provided the best quality for constructing structural models of four NAD-domain conformations.

Fig. S6(B) shows the correlation scores between different classification trials (cross-correlation). The plots were used to examine whether almost the same or very similar maps were obtained among classification trials. When very similar maps are obtained in different classification trials, their cross-correlation score becomes significant. For instance, the correlation scores between “Trial A” and “Trial B” indicates that the four NAD-domain conformations commonly appeared in the two trials. The other plots for any pairs of trials also demonstrated that the four NAD-domain conformations were reproduced in each trial.

*Correlation scores in the classification for the steady stage*

For the images of the steady stage, we examined maps from four classification trials (“Trial a”‒“Trial d” in Fig. S7). The plots for the self-correlation scores indicated that there were, at least, four NAD-domain conformations (Fig. S7(A)). The numbers of off-diagonal components greater than 0.8 were small in “Trial a” and “Trial d”, and the “Trial a” provided maps in the highest quality for model construction and the smallest number of images in the “disordered map” category (see Fig. S8). Detailed inspection of “Trial a” maps in PRCM and HLOP suggested the presence of slightly different NAD-domain conformations from those in the initial stage. As well as in the case of the initial stage, maps of the four NAD-domain conformations appeared among the four trials (Fig. S7(B)).

*Populations of images classified into four NAD-domain conformations*

After the classification trials, we divided the maps into five categories, “disordered” and four NAD-domain conformations, CMPX, PRCM, HLOP and PROP. For the initial stage, the populations of images composing each of five categories were similar among the five trials (Fig. S8(A)). The averaged populations among the trials are shown in Fig. 3C in the main text.

In contrast, for the steady stage, the population of images in the disordered, HLOP and PRCM categories varied among the four trials (Fig. S8(B)). The averaged populations among the trials are shown in Fig. 3C in the main text. The sum of the number of images in the disordered, HLOP and PRCM categories were similar. In classification trials, the mixture of the images in correct orientations and those in incorrect orientations cause the disordered maps. When the disordered category in a trial contained a large number of images, images in HLOP and PRCM are mixed in the category and gave unclear maps of the NAD domains and cofactor molecules. Therefore, the number of images in the disordered and/or uninterpretable maps may be one of measures to evaluate whether the classification goes well.

*How did we treat representative NAD-domain conformations from classification trials?*

Potentially, two strategies may be possible to illustrate the NAD-domain conformations. The first is to take an average of maps displaying correlation scores greater than a threshold among the trials. In practice, most of the trials contained ill separated maps with ghost peaks in the solvent region. Those maps may be mixtures of different NAD-domain conformations to be blurred in the NAD domain. Therefore, when averaging well separated maps from a trial and ill separated maps in the other trials, the averaged map is probably a mixture of NAD-conformations.

The second is the selection of a specific trial, which provide maps with low noise levels and suitable for modeling the NAD-domain conformations and cofactor/ligand molecules. In this study, due to a limited period of time and our computational resources, the second strategy may be better than the first. When a large number of classification trials as large as several hundred are possible, we may more rigorously examine each map by the statistical analysis using the null hypothesis.

*NAD-conformations common between the initial and steady stages*

Cross correlation of maps between the classification trials for the initial and steady stages tells us how many NAD-domain conformations were common between the initial and steady stages. High cross-correlation scores suggest the presences of conformations common between the two stages. Fig. S9 shows examples of cross-correlation scores between maps for the initial and steady stages and confirms the appearance of the four NAD-domain conformations, CMPX, PRCM, HLOP and PROP, in both the initial and steady stages.

*Classification using CryoSPARC*

In addition to the classifications using the *Relion* suite, we performed a classification trial for each stage using *cryoSPARC*^4^. Fig. S10 shows the results just after the 3D classification. For the initial stage (Fig. S10(A)), When assuming eight classes, except class 5, map of each class was superimposable onto either of four NAD-domain conformations (CMPX, PRCM, HLOP and PROP). The map of class 5 can be explained as the mixture of PROP and HLOP. For the steady stage, CMPX and PROP conformations were separated, while maps of three classes were in modest resolution and interpreted as mixtures of HLOP and PRCM conformations (Fig. S10(B)).

The population of images in the six categories including the mixtures of NAD-domain conformations are compared with the results from the classification using *Relion* (Fig. S10(C)). Regarding the CMPX and PROP conformations, the populations of images in the initial and steady stages were similar to each other. The sum of the images in the HLOP, PRCM, unknown and disordered map categories may be comparable between classification trials using the two algorithms.

**References**

1. Scheres, S. H. W. Processing of structurally heterogeneous cryo-EM data. *Meth. Enzymol*. **579**, 125–157 (2016).
2. Kimanius, D., Dong, L., Sharov, G., Nakane, T. & Scheres, S.H.W. New tools for automated cryo-EM single-particle analysis in RELION-4.0. *Biochem. J*. **478**, 4169–4185 (2021).
3. Pettersen, E. F., Goddard, T. D., Huang, C. C., Meng, E. C., Couch, G. S., Croll, T. I., Morris, J. H. & Ferrin, T. E. *UCSF ChimeraX*: Structure visualization for researchers, educators, and developers. *Protein Sci*. **30**, 70–82 (2021).
4. Punjani, A., Rubinstein, J. L., Flee, D. J. & Brubaker, M. A. cryoSPARC: algorithms for rapid unsupervised cryo-EM structure determination. *Nat. Methods* **14**, 290-296 (2017).


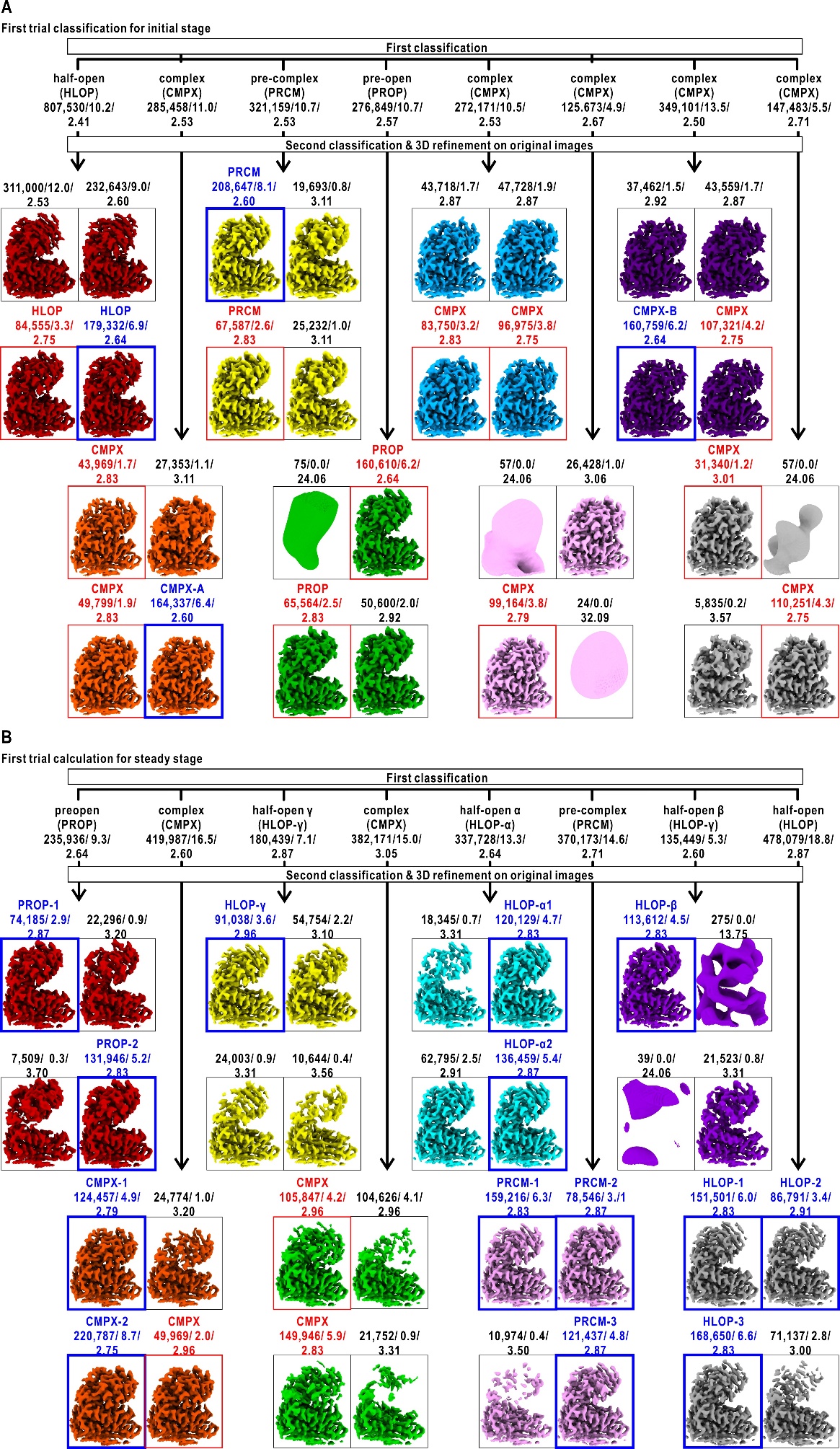


**Supplementary Figure S4**

**Schematic diagram of the two-step focused classification**

Panels (A) and (B) illustrate the results of classification trials for the initial and steady stages, respectively. The label of each subclass indicates the name of the state with the abbreviation in parentheses, the number of images used to construct the map of the subclass, the ratio of the number of images to the total number of images used in the classification in percentage, and the effective resolution estimated using the gold-standard Fourier shell correlation^1^. The subclass map suitable for model building is indicated by a red box with a label of the abbreviated name for NAD-domain conformation. In addition, the representative subclass maps presented in Figs. 3‒6 in the main text are indicated by blue boxes and labels. Panels were prepared using *UCSF Chimera**X*^2^.

**References**

1. Rosenthal, P. B. & Henderson, R. Optimal determination of particle orientation, absolute hand, and contrast loss in single-particle electron microscopy. *J. Mol. Biol*. **333**, 721–745 (2003).
2. Pettersen, E. F., Goddard, T. D., Huang, C. C., Meng, E. C., Couch, G. S., Croll, T. I., Morris, J. H. & Ferrin, T. E. UCSF ChimeraX: Structure visualization for researchers, educators, and developers. *Protein Sci*. **30**, 70–82 (2021).


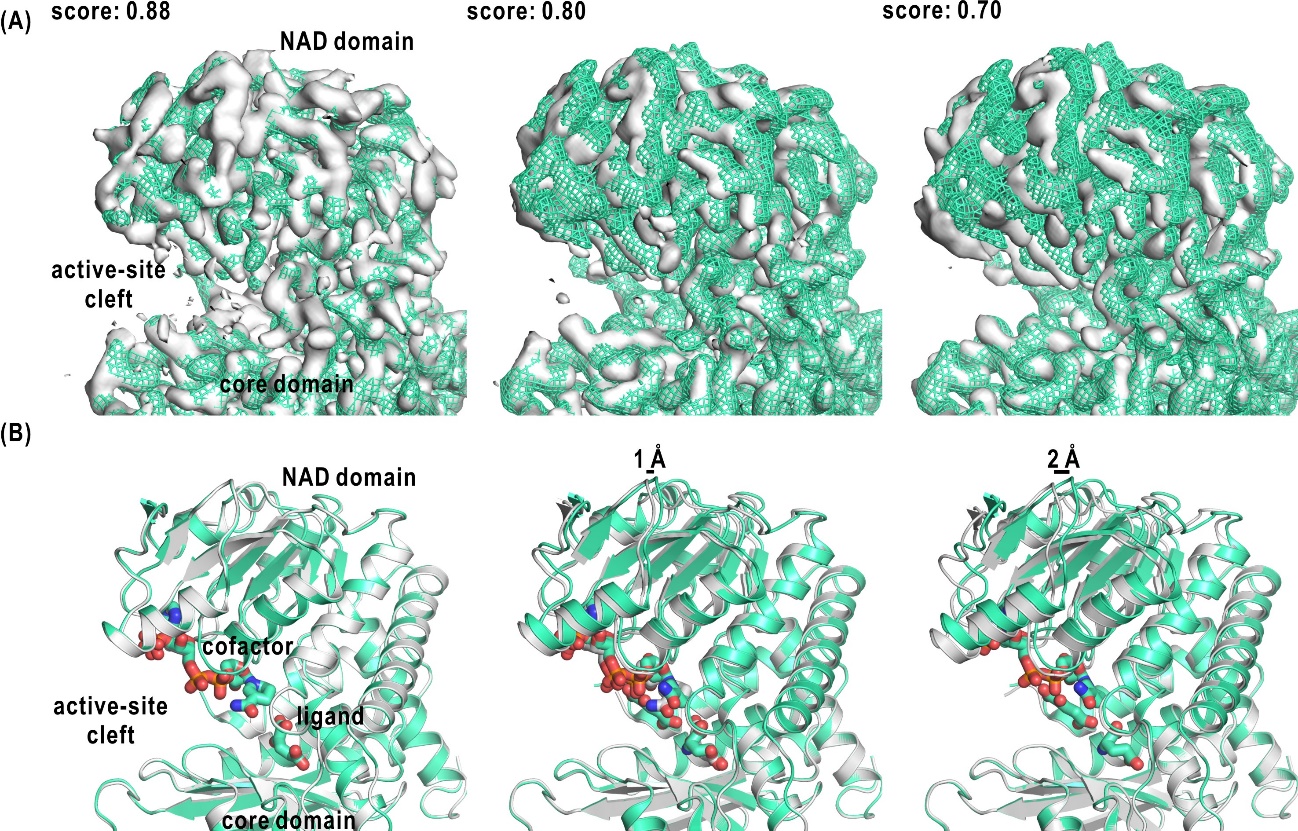


**Supplementary Figure S5**

**Examples of map pairs and their correlation scores**

(A) Comparison of two maps, which give the correlation score labeled at the top left. A map is illustrated by green fish-net and the other is surface-rendered. Maps were contoured at 1.5 standard deviation level from the average. (B) Comparison of the structural models constructed from the maps in panel (A). The structural differences at the top of the NAD domain are labeled. Panels were prepared using *PyMol*^1^.

**Reference**

1. DeLano, W. L. *The PyMOL Molecular Graphics System*, version 1.5.0.1, Schrödinger, LLC, New York.


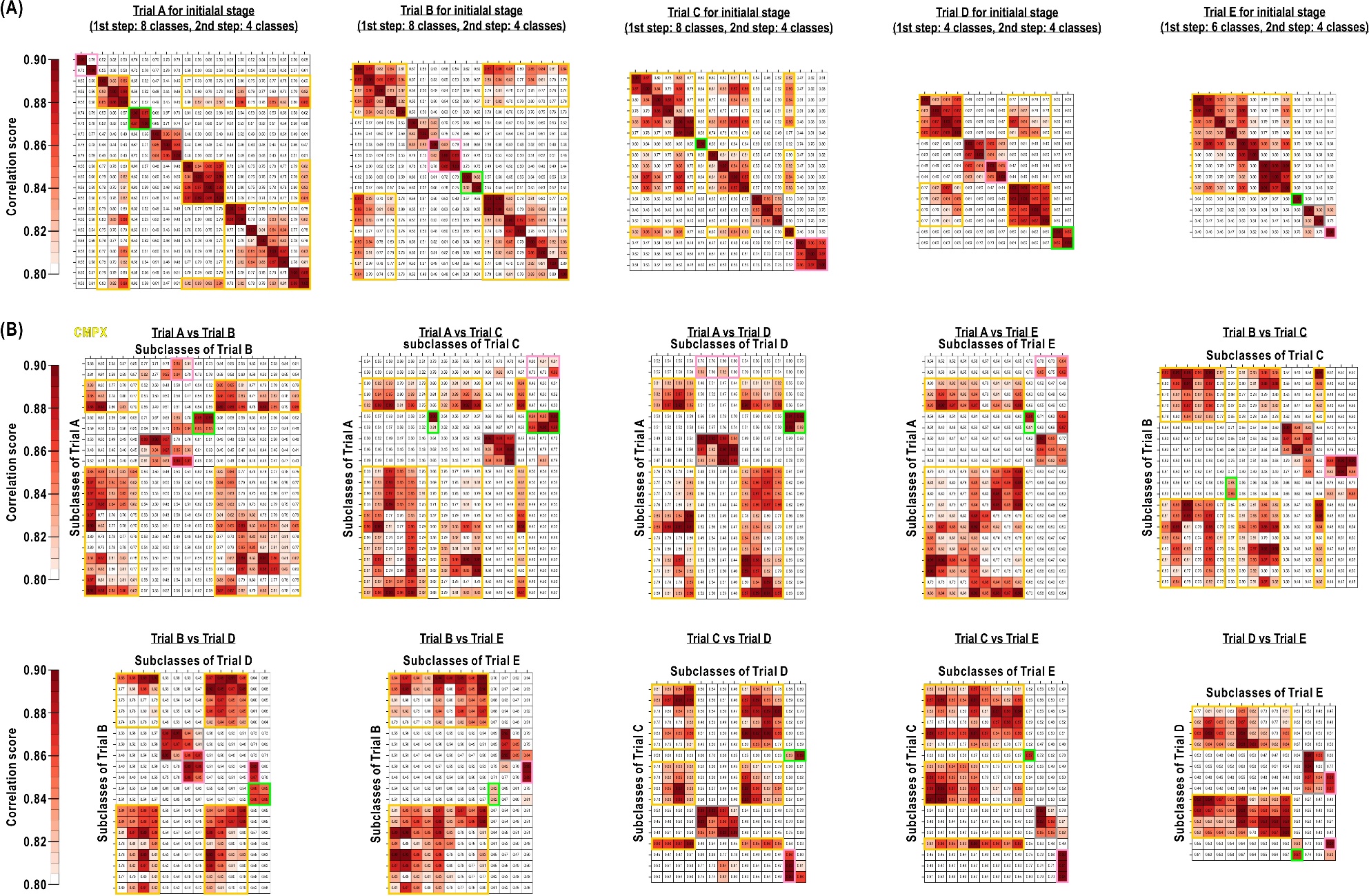


**Supplementary Figure S6**

**Correlation scores of maps in the classification trials for the initial stage**

(A) The self-correlation scores between the maps within each classification trial. The background of each correlation score is colored according to the scheme shown in the left. (B) The cross-correlation scores between maps of two independent classification trials. Almost the same maps between a pair of trials displayed the scores greater than 0.85. Colored box surrounding approximate areas including scores greater than 0.80-0.85 indicates that similar or almost the same maps were obtained between different classification trials. The yellow, green, magenta and gray boxes indicate the CMPX, PRCM, HLOP and PROP conformations, respectively.


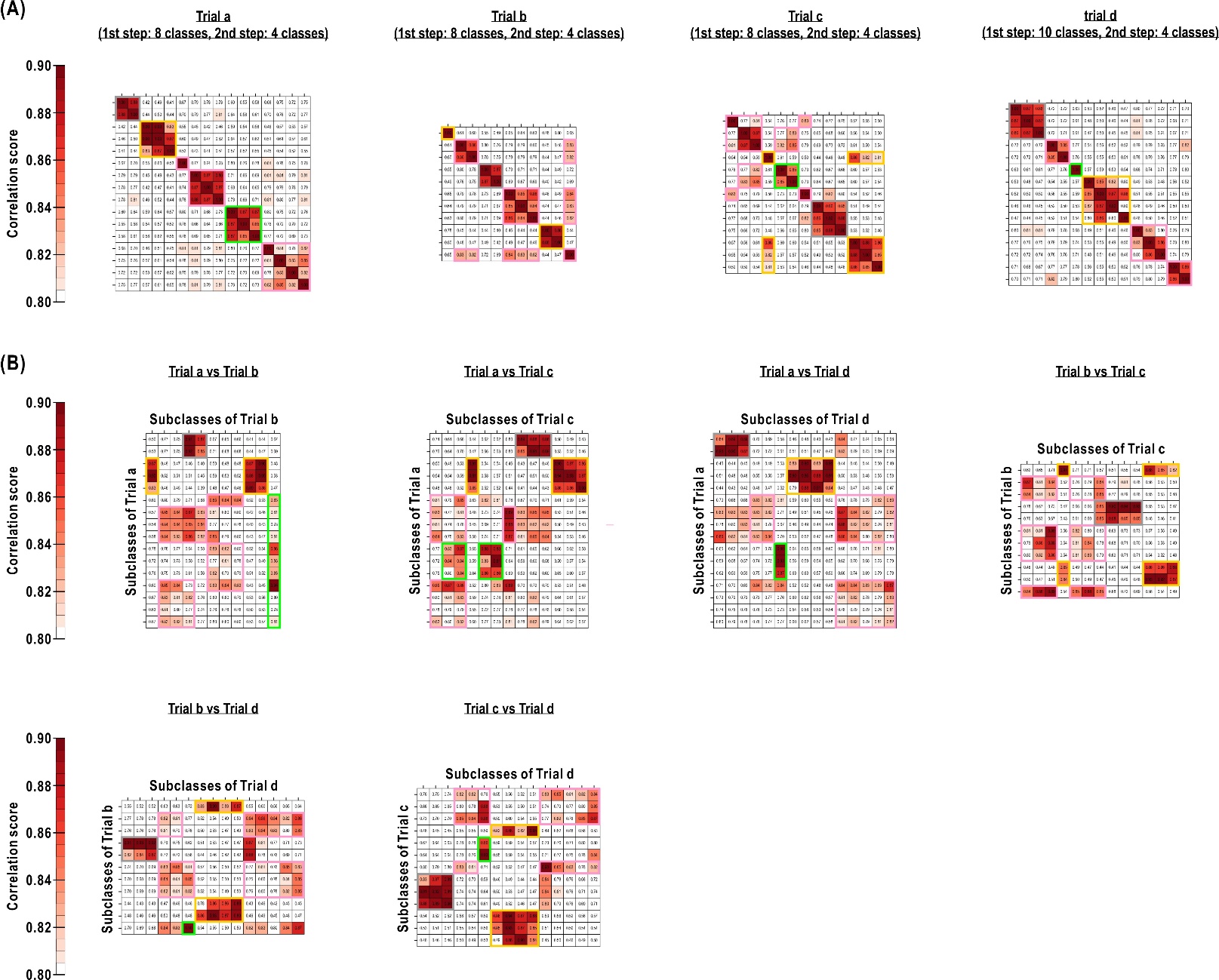


**Supplementary Figure S7**

**Correlation scores of maps in the classification trials for the steady stage**

The self-correlation (panel (A)) and cross-correlation (panel (B)) scores between the maps. The panels are drawn according to the manner in Fig. S6.


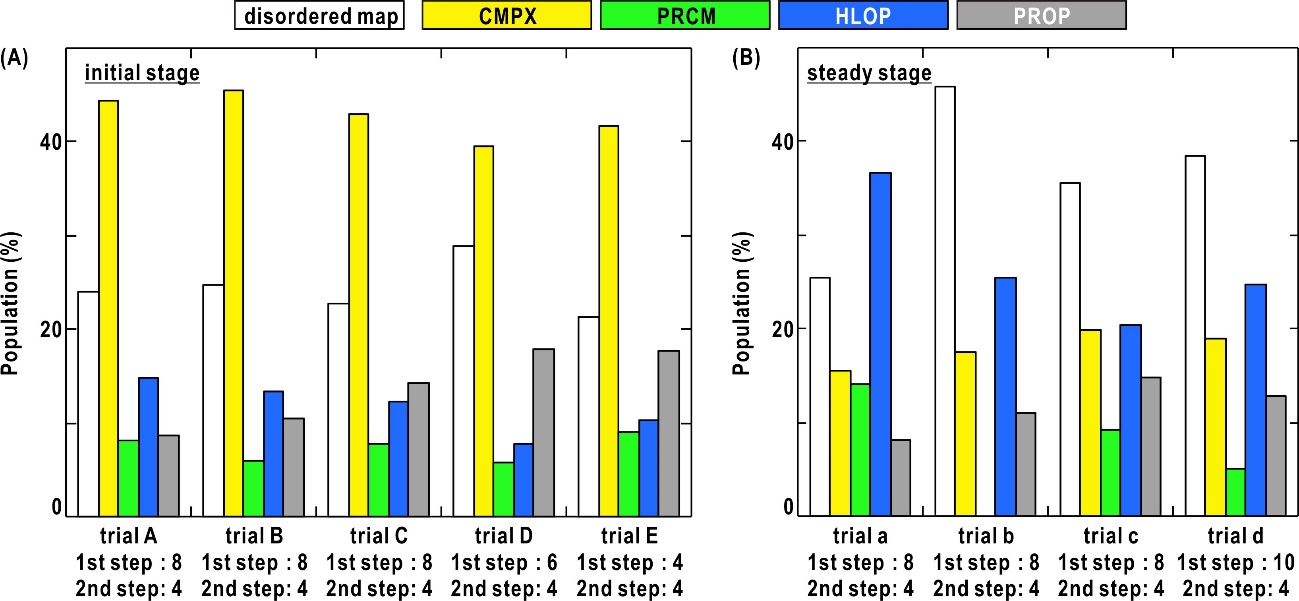


**Supplementary Figure S8**

**Populations of images in five categories**

The populations of images in the five categories at the top in the classification trials for the initial (panel (A)) and steady (panel (B)) stages.

**
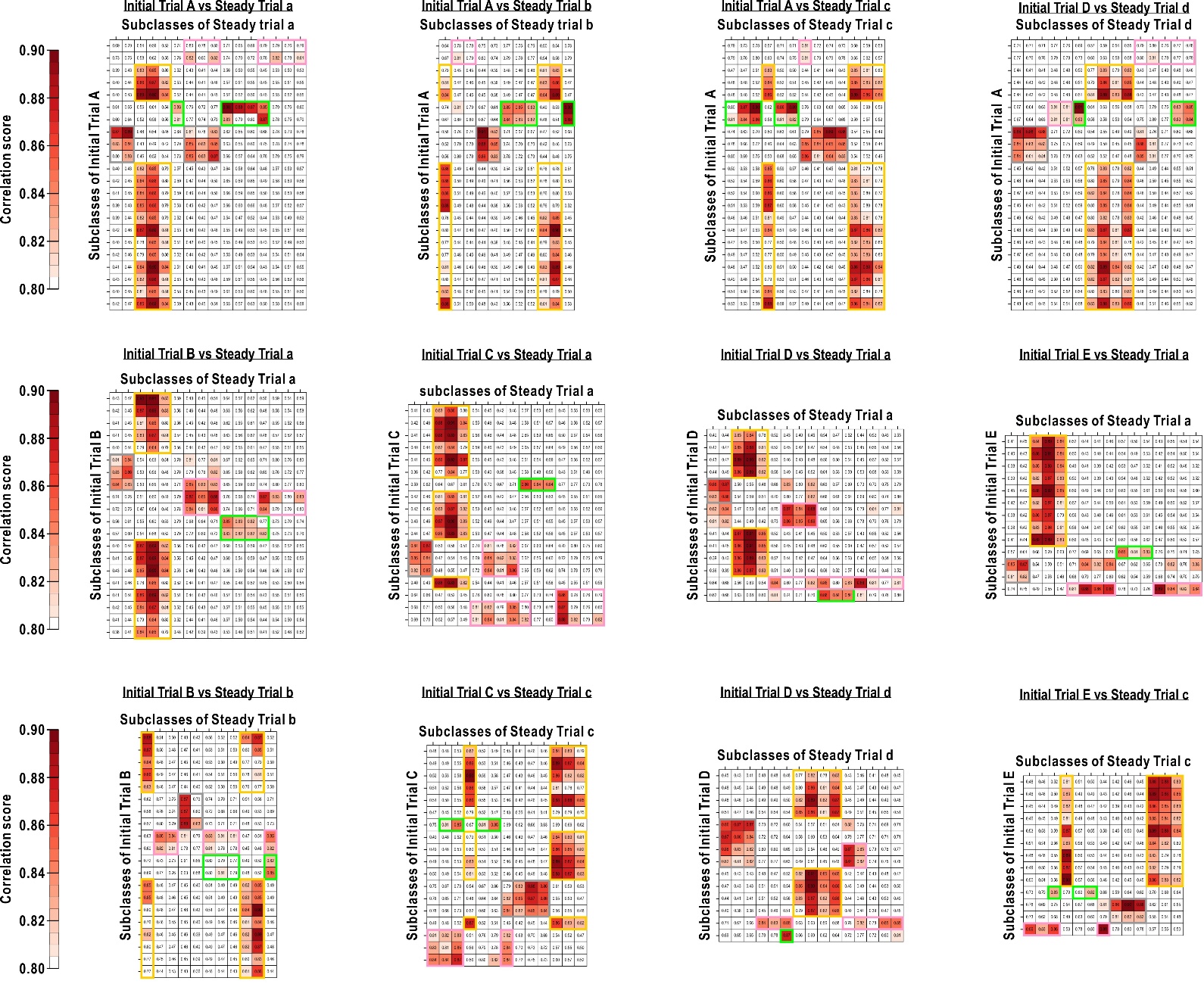
**

**Supplementary Figure S9**

**Correlation scores between classification trials for the initial and steady stages.**

Examples of the cross-correlation scores for pairs of classification trials for the initial and steady stages. The panels are drawn according to the manner in Fig. S6.


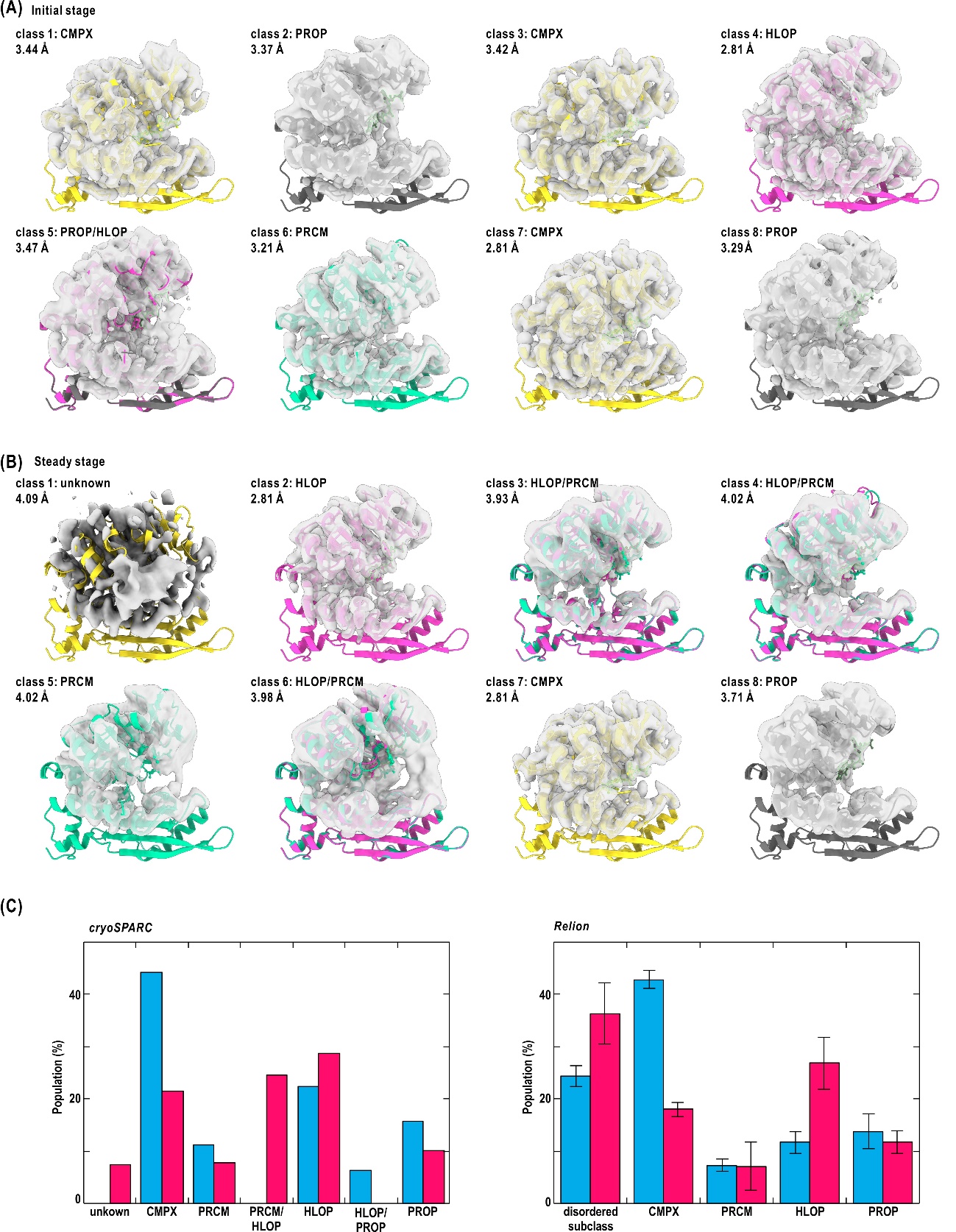


**Supplementary Figure S10**

**Results from a classification trial using *cryoSPARC***

Maps obtained by *cryoSPARC*^1^ under assuming eight classes for the initial (A) and steady (B) stages. The maps were depicted by transparent surfaces. The structural models of CMPX, PRCM, HLOP and PROP conformations were illustrated using the ribbon models colored in yellow, green, magenta and gray, respectively. The *FSC*-resolution is labeled at the top left in each panel. (C) Left panel shows the populations of images in the initial (blue bars) and steady (red bars) stages obtained using *cryoSPARC*. As a reference, the populations obtained using *Relion*^2^ (Fig. 3C in the main text) is shown in the right panel. Panels (A) and (B) were prepared using *ChimeraX*^3^.

References

1. Punjani, A., Rubinstein, J. L., Flee, D. J. & Brubaker, M. A. cryoSPARC: algorithms for rapid unsupervised cryo-EM structure determination. *Nat. Methods* **14**, 290-296 (2017).
2. Kimanius, D., Dong, L., Sharov, G., Nakane, T. & Scheres, S.H.W. New tools for automated cryo-EM single-particle analysis in RELION-4.0. *Biochem. J*. **478**, 4169–4185 (2021).
3. Pettersen, E. F., Goddard, T. D., Huang, C. C., Meng, E. C., Couch, G. S., Croll, T. I., Morris, J. H. & Ferrin, T. E. UCSF ChimeraX: Structure visualization for researchers, educators, and developers. *Protein Sci*. **30**, 70–82 (2021).


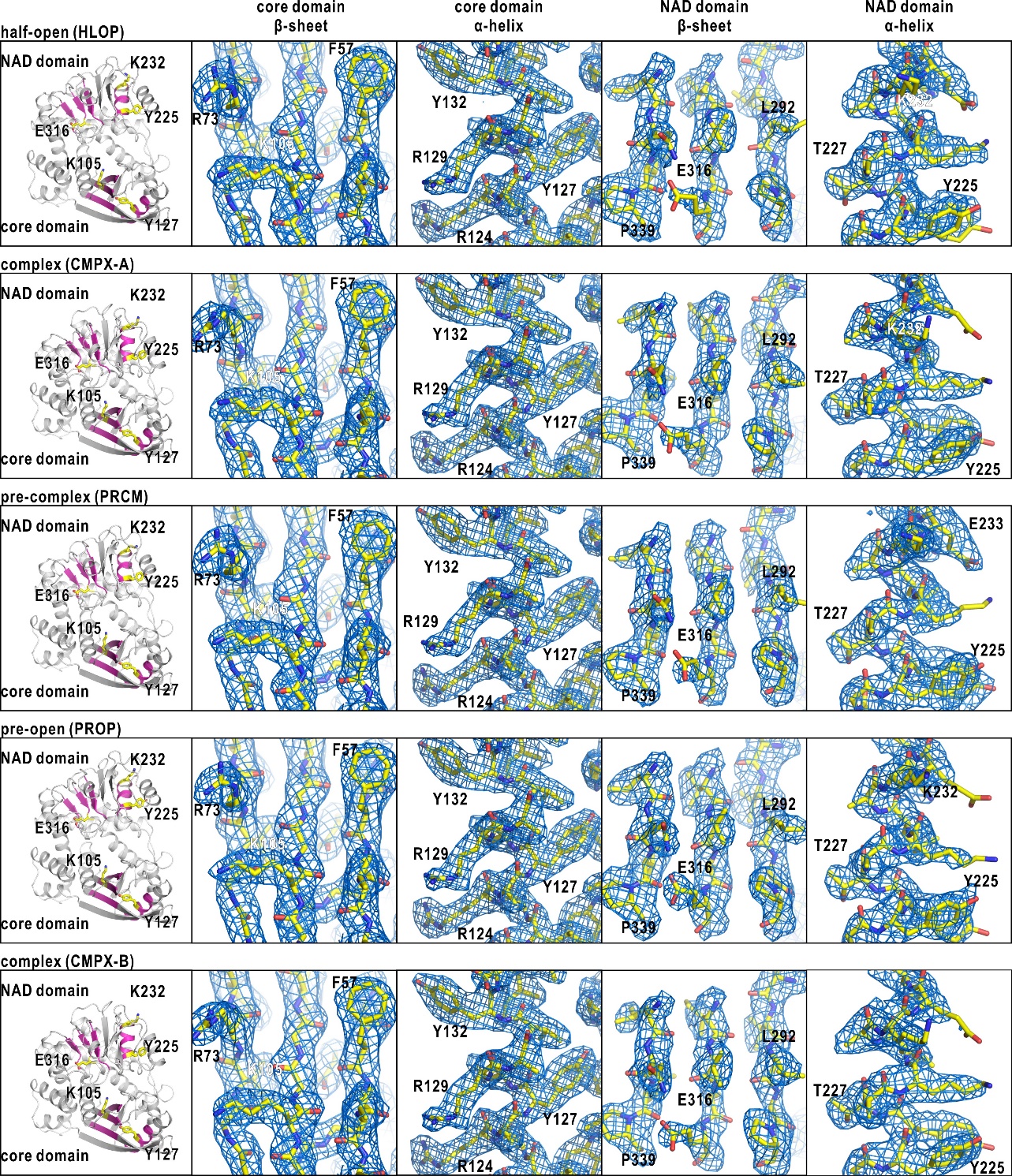


**Supplementary Figure S11**

**Quality of subclass maps of the initial stage.**

Potential maps of representative secondary structures in the core and NAD domains in the selected classification “Trial A”. The panels in the left column illustrate the locations of the secondary structures (magenta) and the sidechains of the landmark residues (yellow) in the subunit. The potential maps were trimmed using *Coot*^1^ and contoured at 3 standard deviation level from the average. All panels were drawn using *PyMol*^2^

**References**

1. Emsley, P., Lohkamp, B., Scott, W. G. & Cowtan, K. Features and Development of *Coot*. *Acta Crystallogr*. D**66**, 486–501 (2010).
2. DeLano, W. L. The *PyMOL* Molecular Graphics System, version 1.5.0.1, Schrödinger, LLC, New York.

**
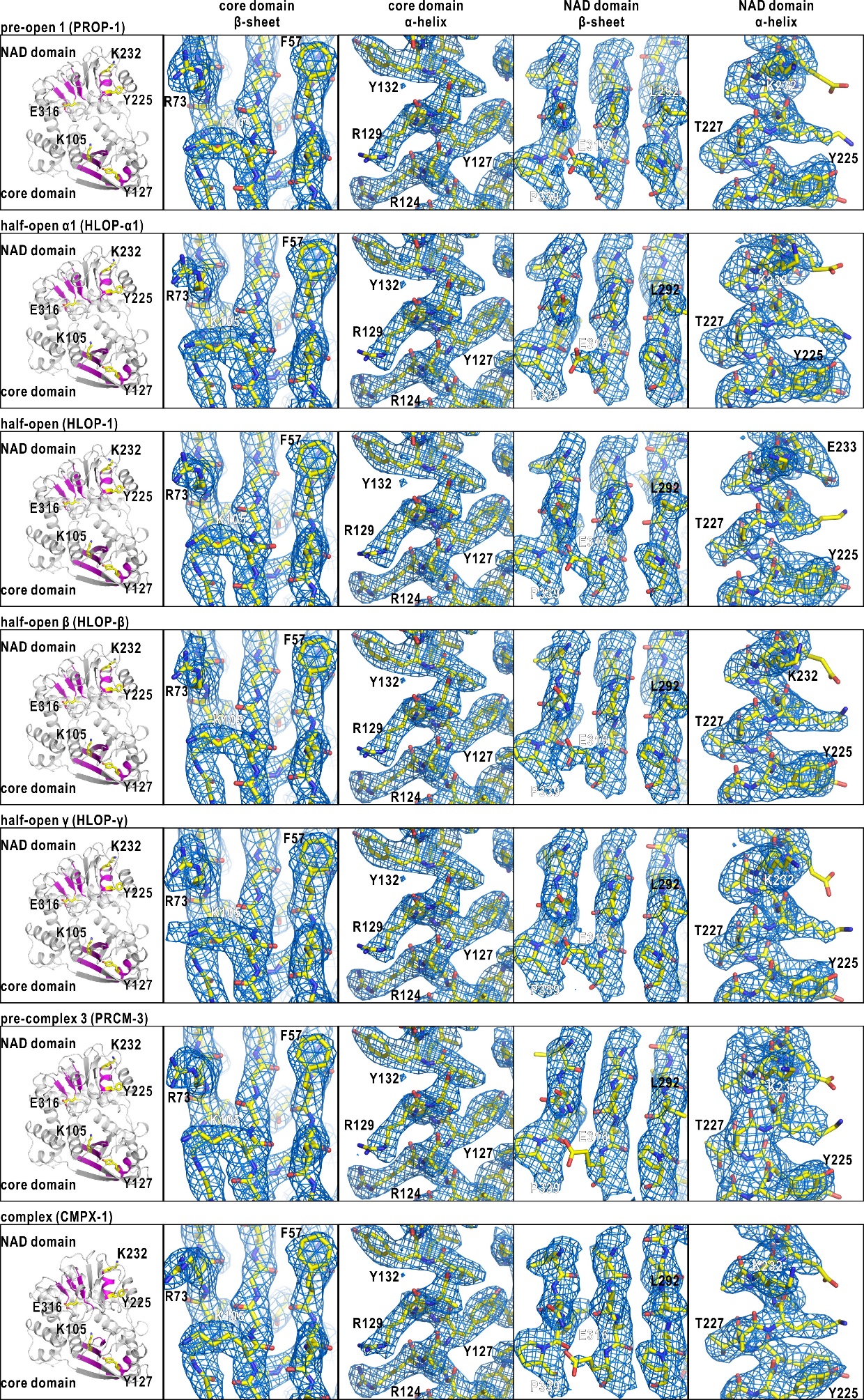
**

**Supplementary Figure S12**

**Quality of subclass maps of the steady stage.**

Potential maps of representative secondary structures in the core and NAD domains in the selected classification “Trial a”. The panels are illustrated in the manner of Fig. S11.

**Supplementary Table S2**

Summary on the resolution of the reconstructed maps used for model building and the stereochemical validation of the constructed structural models in the initial and steady stages.

| Stage /  state | PDB ID | *FSC* resolution (Å) / # of images used | Ramachandran plot  favored/other/outlier (%) | Fatal contacts of non-H atoms |
| --- | --- | --- | --- | --- |
| Initial |  |  |  |  |
| PROP | 8XCO | 2.64 / 160,610 | 92.7 / 7.3 / 0 | none |
| HLOP | 8XCP | 2.64 / 179,332 | 92.4 / 7.5 / 0 | none |
| PRCM | 8XCQ | 2.60 / 208,647 | 94.0 / 7.0 / 0 | none |
| CMPX-A | 8XCR | 2.60 / 164,337 | 94.0 / 6.0 / 0 | none |
| CMPX-B | 8XCS | 2.64 / 160,759 | 93.7 / 6.3 / 0 | none |
| Steady |  |  |  |  |
| PROP-1 | 8XCT | 2.87 / 74,185 | 93.5 / 6.5 / 0 | none |
| PROP-2 | 8XCU | 2.83 / 131,946 | 93.5 / 6.5 / 0 | none |
| HLOP-α1 | 8XCV | 2.83 / 120,129 | 94.7 / 5.3 / 0 | none |
| HLOP-α2 | 8XCW | 2.87 / 136,459 | 93.7 / 5.3 / 0 | none |
| HLOP-β | 8XD0 | 2.83 / 113,612 | 93.7 / 6.3 / 0 | none |
| HLOP-1 | 8XCX | 2.83 / 151,501 | 93.2 / 6.8 / 0 | none |
| HLOP-2 | 8XCY | 2.92 / 86,791 | 93.4 / 6.5 / 0 | none |
| HLOP-3 | 8XCZ | 2.79 / 168,650 | 92.7 / 7.3 / 0 | none |
| HLOP-γ | 8XD1 | 2.92 / 91,038 | 93.4 / 6.5 / 0 | none |
| PRCM-1 | 8XD2 | 2.83 / 159,216 | 93.9 / 6.0 / 0 | none |
| PRCM-2 | 8XD3 | 2.87 / 78,546 | 93.0 / 7.0 / 0 | none |
| PRCM-3 | 8XD4 | 2.87 / 121,437 | 93.0 / 7.0 / 0 | none |
| CMPX-1 | 8XD5 | 2.75 / 220,787 | 94.0 / 6.0 / 0 | none |
| CMPX-2 | 8XD6 | 2.83 / 124,457 | 94.0 / 6.0 / 0 | none |

The validation on the Ramachandran plots and the contacts of non-hydrogen atoms were taken from the results obtained using the validation server of the Protein Data Bank.


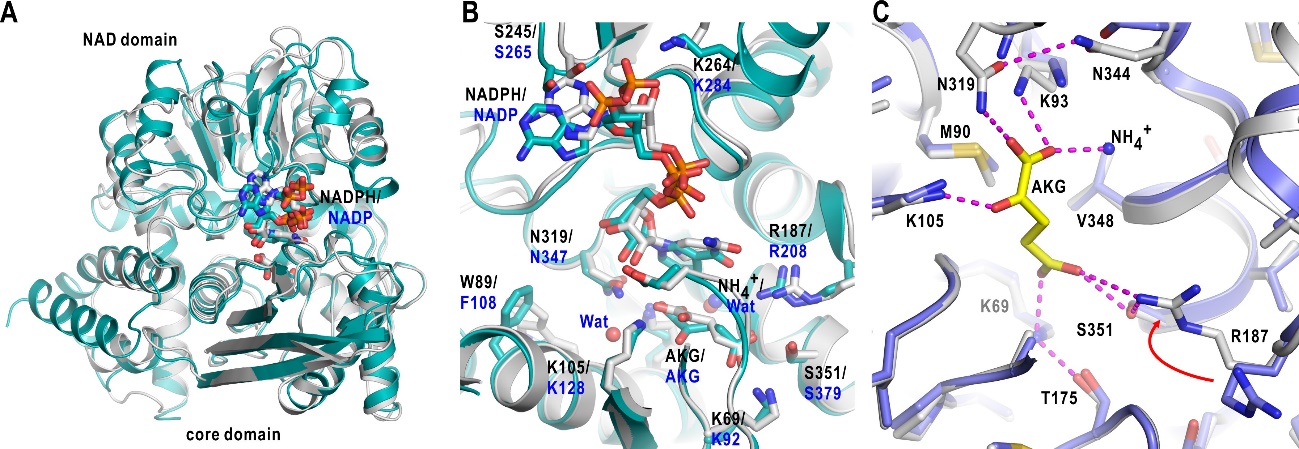


**Supplementary Figure S13**

**Structural comparison of CMPX-2 with the crystal structure of *Corynebacterium glutamicum* GDH in the ternary complex and *Thermococcus profundus* GDH in the unliganded state.**

(A, B) Structural comparison of the CMPX-2 conformation in the steady stage (white colored model) with *Corynebacterium glutamicum* GDH in a non-productive complex with NADP and 2-oxoglutarate (cyan-colored model, the PDB accession code is 5IJZ)^1^. The two structures are optimally superimposed with respect to the secondary structures. Panel (A) presents the overall view of the subunit, and (B) is a magnified view of the active-site cleft. Some groups and amino acid residues are labeled. Black and blue labels are used for CMPX-2 and *C. glutamicum* GDH, respectively. (C) Structural comparison of the ligand binding pocket of CMPX-2 with that in the subunit E of the crystal structure in the unliganded state (blue-colored model, the PDB accession code is 1EUZ)^2^. The red arrow indicates the conformational change of the Arg187 sidechain between CMPX-2 and the unliganded state. The magenta dashed-lines are possible hydrogen bonds with a donor-acceptor distance shorter than 3.4 Å. The panels were prepared using *PyMol*^3^.

**Reference**

1. Son, H. F., Kim, I. K. & Kim, K. J. Structural insights into domain movement and cofactor specificity of glutamate dehydrogenase from *Corynebacterium glutamicum*. *Biochem. Biophys. Res. Commun*. **459**, 387–392 (2015).
2. Nakasako, M., Fujisawa, T., Adachi, S., Kudo, T. & Higuchi, S. Large-scale domain movements and hydration structure changes in the active-site cleft of unligated glutamate dehydrogenase from *Thermococcus profundus* studied by cryogenic X-ray crystal structure analysis and small-angle X-ray scattering. *Biochemistry* **40**, 3069–3079 (2001).
3. DeLano, W. L. The *PyMOL* Molecular Graphics System, version 1.5.0.1, Schrödinger, LLC, New York.

**
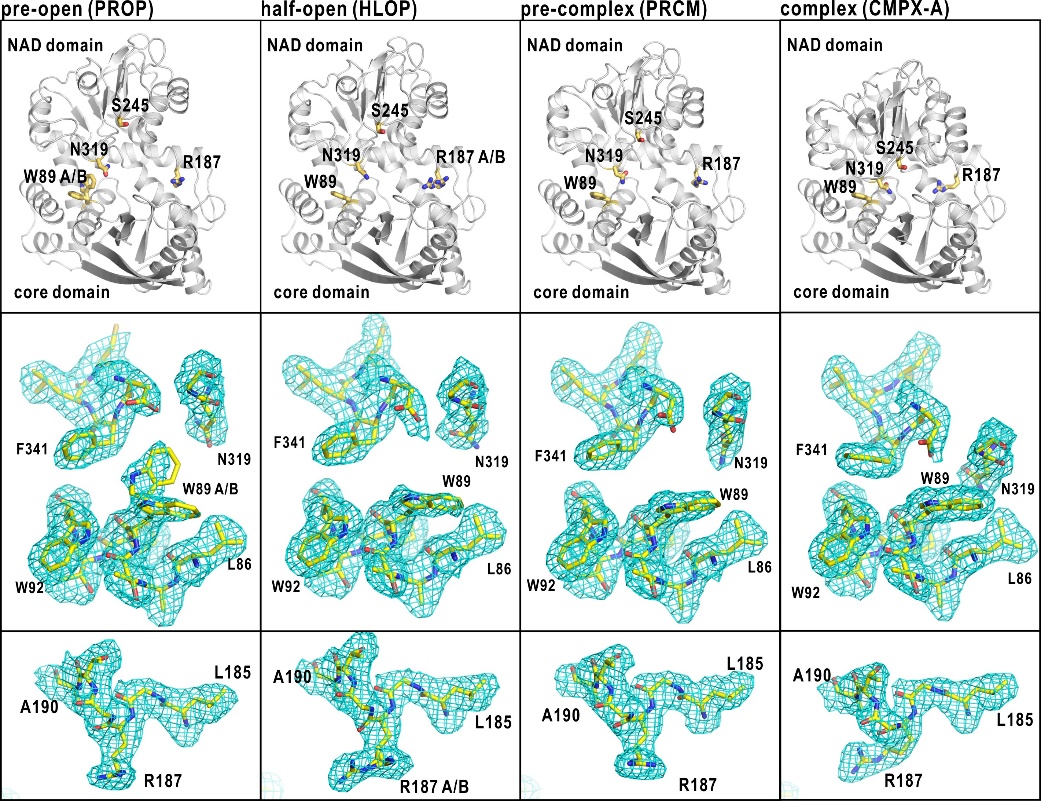
**

**Supplementary Figure S14**

**Maps around Trp89 and Arg187 side chains of subclasses in the initial stage.**

Potential maps around Trp89 and Arg187, the side chains of which displayed alternate conformations depending on the NAD-domain motion. The locations of Trp89 and Arg187 sidechains in the subunit are indicated in the top row. The potential maps around Trp89 and Arg187 are shown in the second and third rows, respectively. The maps were trimmed using *Coot*^1^ and contoured at 3 standard deviation level from the average. All panels were drawn using *PyMol*^2^

**References**

1. Emsley, P., Lohkamp, B., Scott, W. G. & Cowtan, K. Features and Development of *Coot*. *Acta Crystallogr*. D**66**, 486–501 (2010).
2. DeLano, W. L. The *PyMOL* Molecular Graphics System, version 1.5.0.1, Schrödinger, LLC, New York.

**
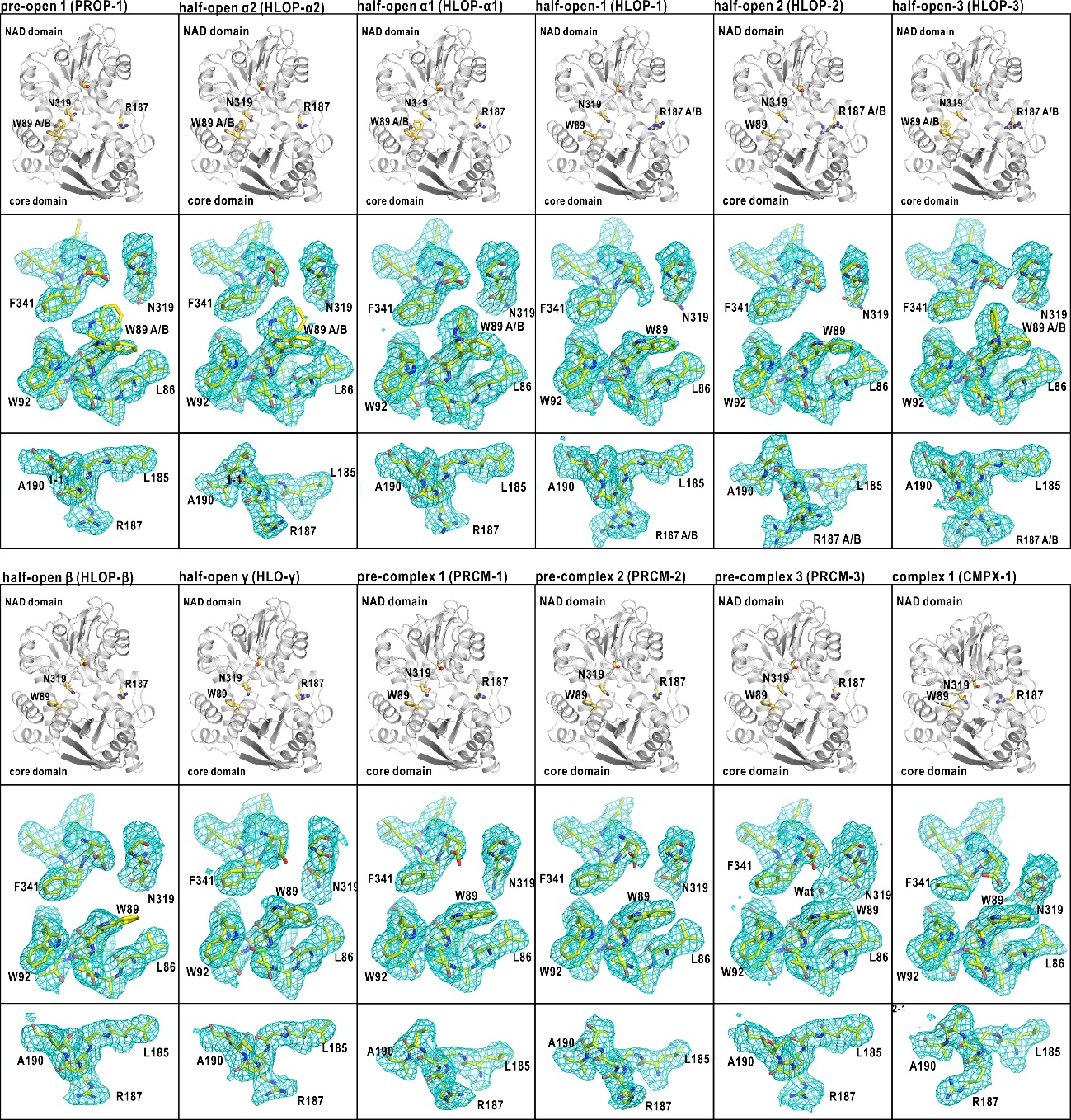
**

**Supplementary Figure S15**

**Maps around Trp89 and Arg187 side chains of subclasses in the steady stage.**

The panels are drawn in the manner in Fig. S14.

**Supplementary Note 3**

*Molecular models of cofactors*

The molecular models of cofactors were placed in the Y-shaped potential maps (Figs. 4-6 in the main text). As described in the main text, there were two types of potential maps. In the first type, maps of whole cofactor molecules appeared. In the second type, only adenosine and pyrophosphate groups were visible. We examined how well the atomic models reproduce the maps using the correlation coefficient^1^, and measured the resolvability of their maps using the *Q*-scores^2^ (Table S3). It should be noted that the *Q*-scores were in correlation with the effective resolution for maps, and the average *Q*-score at a resolution of 2.5 Å was approximately 0.70, as reported^2^.

*Models of ligand and water molecules in and around the ligand-binding pocket*

Molecules occupying the ligand-binding pocket and the flank of cofactor molecules were first modeled by visual inspection of the maps (Figs. S16 and S17). At a local resolution-range of 2.5-2.3 Å, the potential map of a glutamate molecule with the Cα atom in the *sp*^3^-hybridization may be distinguishable from that of 2-oxoglutarate molecule with the C2 atom in the *sp*^2^-hybridization. Glutamate molecules were modeled to maps with bulges corresponding to the nitrogen atoms bonded to the Cα atoms, while maps in planar shapes were modeled as 2-oxoglutarate.

As well as for cofactor models, we examined how well the models reproduced the maps using the correlation coefficient^1^, and measured the resolvability of the maps using the *Q*-scores^2^ (Table S4). In addition, we calculated the correlation coefficients and scores by exchanging each glutamate model by 2-oxoglutarate model, and *vise versa* (Table S4). The models built by visual inspection displayed slightly greater scores than the replaced models.

**References**

1. Afonine, P. V., Klaholz, B. P., Moriarty, N. W., Poon, B. K., Sobolev, O. V., Terwilliger, T. C., Adamsa, P. D., & Urzhumtsev, A. New tools for the analysis and validation of cryo-EM maps and atomic models. *Acta Crystallogr*. D**74**, 814–840 (2018).
2. Pintilie , G., Zhang, K. , Su, Z., Li , S., Schmid, M. F.  & Chiu, W. Measurement of atom resolvability in cryo-EM maps with Q-scores. *Nat. Methods* **17**, 328-344 (2020).

**Supplementary Table S3.**

The correlation coefficients and *Q*-scores between cofactor and the potential maps for the structural models in the initial and steady stages.

|  | Figure | Molecule^a^ | *CC*_msk_^b^ | *CC*_box_^b^ | *CC*_pks_^b^ | *CC*_vol_^b^ | *Q*_avg_^c^ | *Q*_worst_^c^ |
| --- | --- | --- | --- | --- | --- | --- | --- | --- |
| Initial stage | |  |  |  |  |  |  |  |
| HLOP | Fig. 6A | ADPP | 0.65 | 0.78 | 0.79 | 0.69 | 0.62 | 0.13 |
| CMPX-A | Fig. 4A | NAP | 0.87 | 0.87 | 0.88 | 0.87 | 0.75 | 0.46 |
| PRCM^d^ | Fig. 6A  Fig. S16 | ADPP | 0.78 | 0.87 | 0.87 | 0.79 | 0.70 | 0.10 |
|  |  | NDP | 0.74 | 0.83 | 0.84 | 0.77 | 0.67 | ‒0.14 |
| PROP | Fig. 5A | NDP | 0.80 | 0.88 | 0.88 | 0.82 | 0.71 | 0.17 |
| CMPX-B | Fig. 4B | NDP | 0.79 | 0.82 | 0.83 | 0.79 | 0.70 | ‒0.36 |
| Steady stage | |  |  |  |  |  |  |  |
| PROP-1 | Fig. 5B | NDP | 0.83 | 0.85 | 0.86 | 0.84 | 0.73 | 0.18 |
| PROP-2 | Fig. 5E | NDP | 0.81 | 0.84 | 0.85 | 0.83 | 0.72 | 0.35 |
| CMPX-2 | Fig. 4D | NDP | 0.86 | 0.83 | 0.85 | 0.86 | 0.73 | ‒0.08 |
| CMPX-1 | Fig. 4A | NAP | 0.90 | 0.88 | 0.89 | 0.90 | 0.77 | 0.40 |
| HLOP-γ | Fig. 6C | ADPP | 0.75 | 0.87 | 0.87 | 0.77 | 0.71 | 0.48 |
| HLOP-α2 | Fig. 6C | ADPP | 0.86 | 0.89 | 0.90 | 0.87 | 0.75 | 0.45 |
| HLOP-α1 | Fig. 5C | NDP | 0.77 | 0.85 | 0.85 | 0.80 | 0.67 | -0.25 |
| PRCM-1 | Fig. 6D | ADPP/GGL | 0.66 | 0.8 | 0.81 | 0.69 | 0.60 | 0.02 |
|  |  | NDP | 0.58 | 0.72 | 0.72 | 0.61 | 0.71 | 0.48 |
| PRCM-2 | Fig. 6D | ADPP | 0.85 | 0.88 | 0.89 | 0.86 | 0.75 | 0.34 |
| PRCM-3^d^ | Fig. 6D  Fig. S16 | ADPP/GGL | 0.74 | 0.82 | 0.83 | 0.76 | 0.67 | 0.07 |
|  |  | NDP | 0.58 | 0.67 | 0.68 | 0.61 | 0.58 | ‒0.22 |
| HLOP-β | Fig. 5D | ADPP | 0.78 | 0.83 | 0.84 | 0.8 | 0.69 | ‒0.03 |
| HLOP-1 | Fig. 6B | ADPP | 0.72 | 0.82 | 0.83 | 0.75 | 0.70 | 0.23 |
| HLOP-2 | Fig. 6B | ADPP | 0.65 | 0.73 | 0.74 | 0.68 | 0.58 | ‒0.18 |
| HLOP-3 | Fig. 6B | ADPP | 0.64 | 0.75 | 0.76 | 0.66 | 0.61 | ‒0.12 |

^a^ The abbreviations of molecular names: glutamate; GGL, NADPH; NDP, NADP; NAP, and adenosine-pyrophosphate group of NADP/NADPH; ADPP.

^b^ The correlation coefficient^1^ between two potential maps, $\rho_{1}\left(\text{n}\right)$and $\rho_{2}\left(\text{n}\right)$on the same grid $\left\{\text{n}\right\}$, was calculated according to the proposed protocols^1^ using the following equation as

$CC\left(\rho_{1},\rho_{2}\right)=\sum_{\text{n}} \left[\rho_{1}\left(\text{n}\right)-\left\langle\rho_{1}\right\rangle\right]\left[\rho_{2}\left(\text{n}\right)-\left\langle\rho_{2}\right\rangle\right]/\left\{\sqrt{\sum_{\text{n}} \left[\rho_{1}\left(\text{n}\right)-\left\langle\rho_{1}\right\rangle\right]^{2}}\sqrt{\sum_{\text{n}} \left[\rho_{2}\left(\text{n}\right)-\left\langle\rho_{2}\right\rangle\right]^{2}}\right\}$.

*CC*_msk_, *CC*_box_, *CC*_pks_, and *CC*_vol_ were calculated for grid points belonging to a generated molecular mask, all grid points in a targeted box, the highest value grid points in the model and potential maps and grid points with the highest values of the model map, respectively.

^c^ The *Q*-score^2^ is defined calculated as a correlation between two vectors using the normalized about-the-mean cross-correlation formula as

$Q=\left[\left\langle\mathbf{u}-\mathbf{u}_{\text{mean}}\right\rangle\left\langle\mathbf{v}-\mathbf{v}_{\text{mean}}\right\rangle\right]/\left[\left|\mathbf{u}-\mathbf{u}_{\text{mean}}\right|\left|\mathbf{v}-\mathbf{v}_{\text{mean}}\right|\right]$,

where **u** contains map values at points around the atom, and **v** contains values obtained from the reference Gaussian of the model.

^d^ For PRCM in the initial and steady stage, the maps of cofactor molecules are interpreted as an adenosine-pyrophosphate group of a cofactor molecule accompanying a glutamate molecule or a whole cofactor molecule as depicted in Fig. S16. In this study, we selected the former case, because of the scores were slightly better than the latter.

**References**

1. Afonine, P. V., Klaholz, B. P., Moriarty, N. W., Poon, B. K., Sobolev, O. V., Terwilliger, T. C., Adamsa, P. D., & Urzhumtsev, A. New tools for the analysis and validation of cryo-EM maps and atomic models. *Acta Crystallogr*. D**74**, 814–840 (2018).
2. Pintilie , G., Zhang, K. , Su, Z., Li , S., Schmid, M. F.  & Chiu, W. Measurement of atom resolvability in cryo-EM maps with Q-scores. *Nat. Methods* **17**, 328-344 (2020).

**Supplementary Table S4.**

The correlation coefficients and *Q*-scores between molecular models and the potential maps in and around the ligand-binding pocket for the structural models in the initial and steady stage.

|  | Figure | Molecule^a^ | *CC*_msk_ | *CC*_box_ | *CC*_pks_ | *CC*_vol_ | *Q*_avg_ | *Q*_worst_ |
| --- | --- | --- | --- | --- | --- | --- | --- | --- |
| Initial stage | |  |  |  |  |  |  |  |
| HLOP | Fig. 6A | GGL | 0.69 | 0.77 | 0.79 | 0.73 | 0.72 | 0.55 |
|  |  | AKG | 0.66 | 0.76 | 0.76 | 0.67 | 0.66 | 0.40 |
| CMPX-A | Fig. 4A | GGL | 0.71 | 0.66 | 0.69 | 0.71 | 0.70 | 0.10 |
|  |  | H_2_O | 0.77 | 0.46 | 0.48 | 0.77 | 0.90 | 0.90 |
| PRCM | Fig. 6A | GGL | 0.70 | 0.73 | 0.76 | 0.73 | 0.63 | 0.09 |
|  |  | AKG | 0.75 | 0.75 | 0.77 | 0.75 | 0.68 | 0.12 |
|  |  | GGL | 0.83 | 0.88 | 0.88 | 0.85 | 0.79 | 0.69 |
|  |  | AKG | 0.78 | 0.88 | 0.88 | 0.78 | 0.77 | 0.61 |
| PROP | Fig. 5A | 2 H_2_O | 0.62 | 0.8 | 0.82 | 0.64 | 0.82 | 0.81 |
| CMPX-B | Fig. 4B | AKG | 0.72 | 0.66 | 0.66 | 0.73 | 0.74 | 0.32 |
|  |  | NH_4_ | 0.56 | 0.68 | 0.68 | 0.58 | 0.81 | 0.81 |
| Steady stage | |  |  |  |  |  |  |  |
| PROP-1 | Fig. 5B | 2 H_2_O | 0.71 | 0.49 | 0.52 | 0.72 | 0.87 | 0.87 |
| PROP-2 | Fig. 5E | AKG | 0.59 | 0.68 | 0.68 | 0.59 | 0.65 | 0.35 |
|  |  | GGL | 0.54 | 0.64 | 0.66 | 0.59 | 0.58 | ‒0.30 |
|  |  | GGL | 0.58 | 0.16 | 0.18 | 0.53 | 0.64 | 0.28 |
|  |  | AKG | 0.56 | 0.14 | 0.15 | 0.56 | 0.63 | 0.25 |
| CMPX-B | Fig. 4D | AKG | 0.77 | 0.56 | 0.58 | 0.75 | 0.75 | 0.58 |
|  |  | AKG | 0.61 | 0.48 | 0.49 | 0.63 | 0.84 | 0.75 |
|  |  | NH_4_ | 0.54 | 0.22 | 0.24 | 0.62 | 0.78 | 0.78 |
| CMPX-A | Fig. 4C | GGL | 0.69 | 0.53 | 0.58 | 0.68 | 0.74 | 0.53 |
|  |  | H_2_O | 0.62 | -0.12 | -0.1 | 0.56 | 0.75 | 0.75 |
| HLOP-α2 | Fig. 6C | H_2_O | 0.74 | 0.78 | 0.80 | 0.73 | 0.88 | 0.87 |
| HLOP-α1 | Fig. 5C | AKG | 0.72 | 0.76 | 0.78 | 0.69 | 0.73 | 0.54 |
|  |  | GGL | 0.67 | 0.73 | 0.75 | 0.70 | 0.71 | 0.29 |
| PRCM-1 | Fig. 6D | GGL | 0.82 | 0.85 | 0.87 | 0.84 | 0.78 | 0.62 |
|  |  | AKG | 0.81 | 0.84 | 0.86 | 0.81 | 0.76 | 0.59 |
| PRCM-2 | Fig. 6D | GGL | 0.64 | 0.49 | 0.52 | 0.61 | 0.66 | 0.26 |
|  |  | GLU | 0.59 | 0.46 | 0.47 | 0.59 | 0.61 | 0.24 |
| PRCM-3 | Fig. 6D | GGL | 0.81 | 0.87 | 0.88 | 0.83 | 0.80 | 0.69 |
|  |  | AKG | 0.77 | 0.85 | 0.87 | 0.77 | 0.75 | 0.52 |
|  |  | HOH | 0.81 | 0.81 | 0.84 | 0.82 | 0.89 | 0.89 |
| HLOP-β | Fig. 5D | AKG | 0.62 | 0.35 | 0.37 | 0.62 | 0.72 | 0.32 |
|  |  | GGL | 0.60 | 0.39 | 0.43 | 0.58 | 0.66 | 0.38 |
| HLOP-1 | Fig. 6B | GGL | 0.72 | 0.73 | 0.76 | 0.73 | 0.74 | 0.49 |
|  |  | AKG | 0.71 | 0.71 | 0.73 | 0.71 | 0.70 | 0.44 |
|  |  | HOH | 0.86 | 0.65 | 0.69 | 0.85 | 0.92 | 0.92 |
| HLOP-2 | Fig. 6B | GGL | 0.57 | 0.49 | 0.52 | 0.56 | 0.62 | ‒0.28 |
|  |  | AKG | 0.54 | 0.48 | 0.52 | 0.55 | 0.57 | ‒0.49 |
| HLOP-3 | Fig. 6B | GGL | 0.69 | 0.56 | 0.64 | 0.68 | 0.78 | 0.63 |
|  |  | AKG | 0.60 | 0.49 | 0.52 | 0.6 | 0.68 | 0.29 |

^a^ The abbreviations of molecular names: glutamate; GGL and 2-oxoglutarate; AKG.


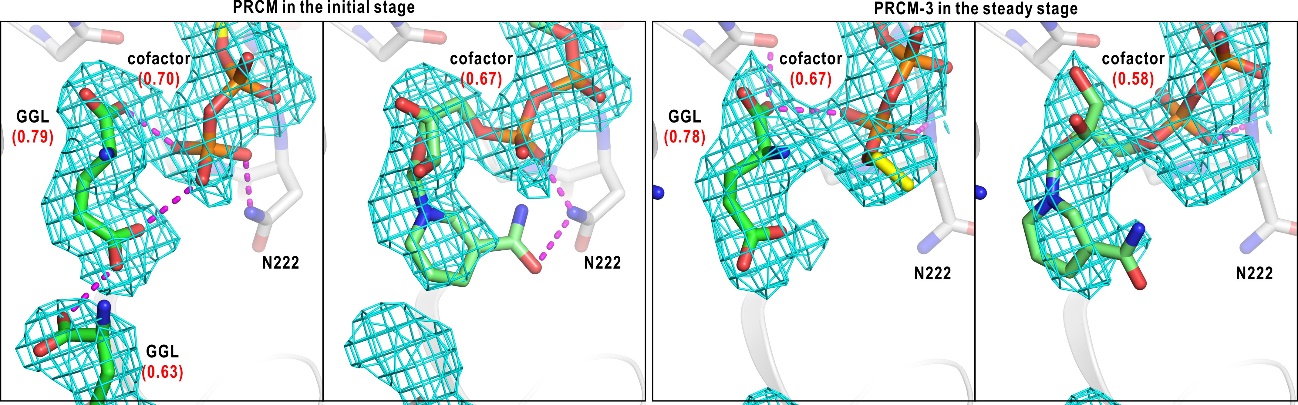


**Supplementary Figure S16**

**Magnified views maps and fitted models at the cofactor-binding site in PRCM**

Panels compare the two types of models fitted to the maps at the cofactor binding site in the PRCM conformations in the initial and steady stages. In the left panel, the map at the flank of the pyrophosphate group in cofactor map was interpreted as one glutamate molecule. In the right panel, the map was interpreted as the ribose-nicotinamide group and putatively NADPH molecule is fitted to the map. Potential maps were trimmed using *Coot*^1^ and contoured at 3 standard deviation level from the average. The *Q*-score^2^ values of cofactor and ligand molecules (Tables S3 and S4) are shown in the parentheses under the abbreviated molecular names. The magenta dashed-lines indicate possible hydrogen bonds (donor-acceptor distance < 3.4 Å). GGL is the abbreviated name of glutamate. All panels were drawn using *PyMol*^3^.

**References**

1. Emsley, P., Lohkamp, B., Scott, W. G. & Cowtan, K. Features and Development of *Coot*. *Acta Crystallogr*. D**66**, 486–501 (2010).
2. Pintilie , G., Zhang, K. , Su, Z., Li , S., Schmid, M. F.  & Chiu, W. Measurement of atom resolvability in cryo-EM maps with Q-scores. *Nat. Methods* **17**, 328-344 (2020).
3. DeLano, W. L. The *PyMOL* Molecular Graphics System, version 1.5.0.1, Schrödinger, LLC, New York.


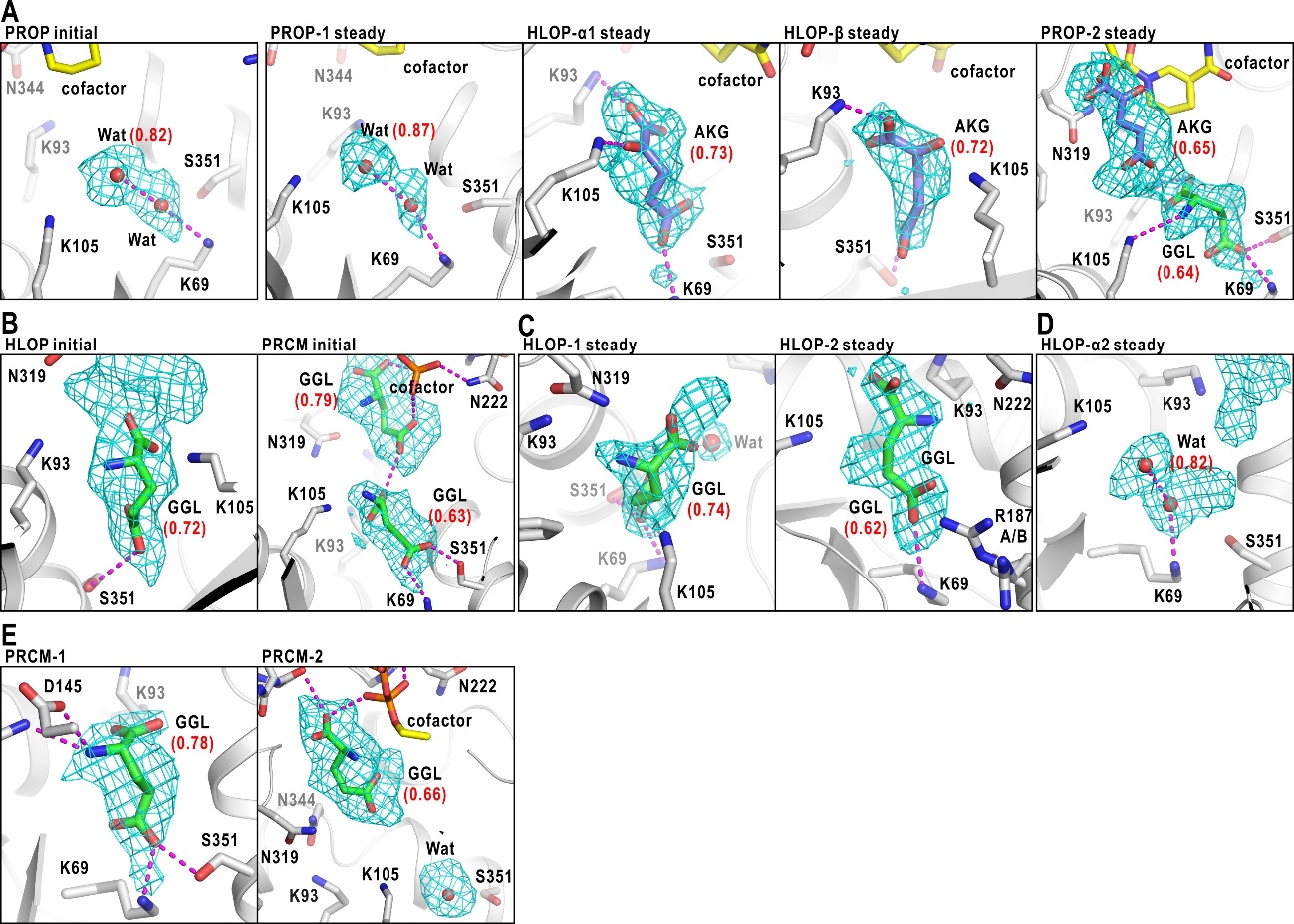


**Supplementary Figure S17**

**Magnified views maps and fitted models in the ligand-binding pocket.**

Potential maps were trimmed using *Coot*^1^ and contoured at 2-3 standard deviation level from the average. The *Q*-score^2^ values of ligand and solvent molecules (Table S4) are shown in the parentheses under the abbreviated molecular names. The magenta dashed-lines indicate possible hydrogen bonds (donor-acceptor distance < 3.4 Å). The abbreviated names of glutamate and 2-oxoglutarate are GGL and AKG, respectively. All panels were drawn using *PyMol*^3^.

**References**

1. Emsley, P., Lohkamp, B., Scott, W. G. & Cowtan, K. Features and Development of *Coot*. *Acta Crystallogr*. D**66**, 486–501 (2010).
2. Pintilie , G., Zhang, K. , Su, Z., Li , S., Schmid, M. F.  & Chiu, W. Measurement of atom resolvability in cryo-EM maps with Q-scores. *Nat. Methods* **17**, 328-344 (2020).
3. DeLano, W. L. The *PyMOL* Molecular Graphics System, version 1.5.0.1, Schrödinger, LLC, New York.


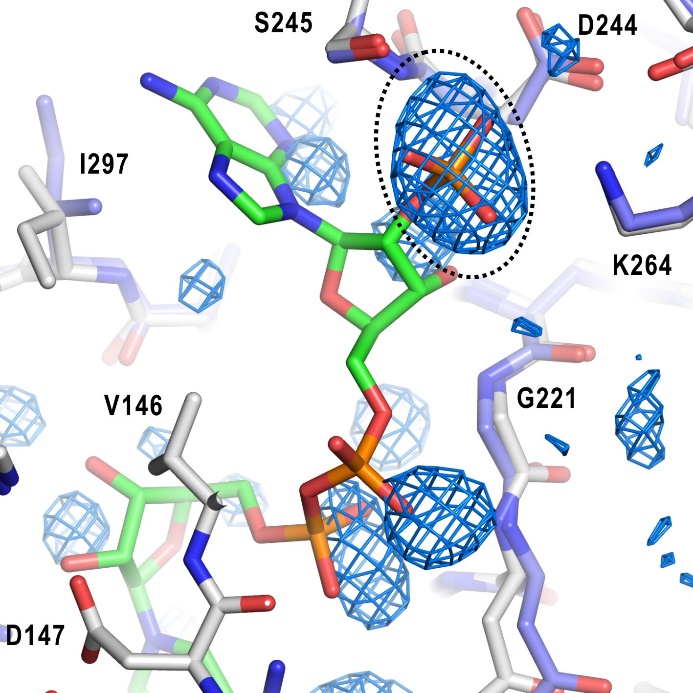


**Supplementary Figure S18**

**Hydration structures excluded in the association of cofactor and ligand molecules.**

Distribution of solvent molecules in subunit E in the crystal structure of unliganded GDH^1,2^ (stick models with blue carbon atoms) around the NADP-binding sites of CMPX (white color). The carbon atoms comprising the NADPH model are colored in green. The blue fishnets are the omit-difference Fourier electron density maps of hydration water molecules and a sulfate ion in subunit E of the crystal structure were calculated at a resolution of 1.8 Å and contoured at 3.5 standard deviation level from the average. The dotted circle indicates the sulfate-ion binding-site in the crystal structure. The phosphate group attached to the ribose group occupied the sulfate-ion binding-site. The cofactor molecule excluded 11 hydration water molecules, which hydrated the crevice between the mainchains of Tyr220 and Ala296 in the unliganded state. This panel was drawn using *PyMol*^3^.

**References**

1. Nakasako, M., Fujisawa, T., Adachi, S., Kudo, T. & Higuchi, S. Large-scale domain movements and hydration structure changes in the active-site cleft of unligated glutamate dehydrogenase from *Thermococcus profundus* studied by cryogenic X-ray crystal structure analysis and small-angle X-ray scattering. *Biochemistry* **40**, 3069–3079 (2001).
2. Sato, K., Oide, M. & Nakasako, M. Prediction of hydrophilic and hydrophobic hydration structure of protein by neural network optimized using experimental data. *Scie. Rep*. **13**, 2183 (2023).
3. DeLano, W. L. The *PyMOL* Molecular Graphics System, version 1.5.0.1, Schrödinger, LLC, New York.


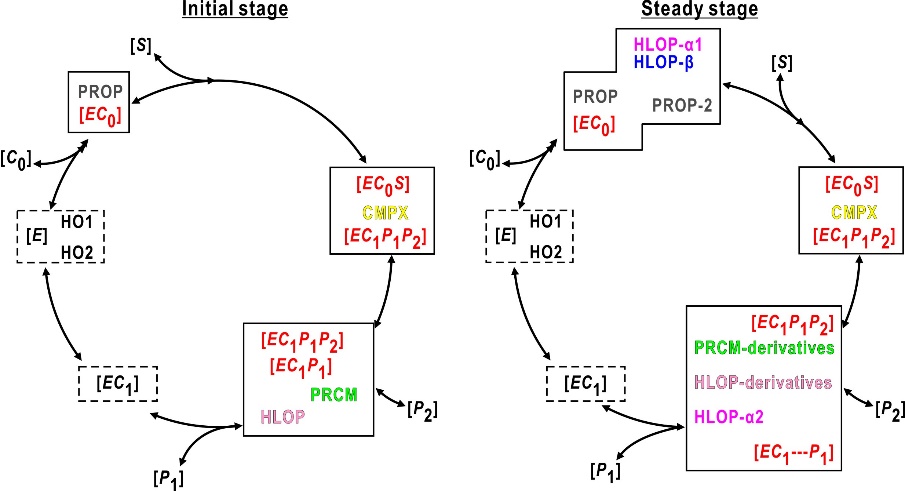


**Supplementary Figure S19**

Hypothetical reaction cycles for the initial (pane A) and steady (B) stages, under assuming that the nicotinamide group is more rigid in NADP than NADPH. The observed states (colored in red) in Fig. 1D of the main text are indicated by solid boxes, while missed states by dashed boxes. The observed conformations are shown in the coloring scheme in Fig. 3D in the main text. [*EC*_1_···*P*_1_] represents the state, where NADPH and 2-oxoglutarate molecules are still bound to the incompletely closed active-site cleft.
